# Engineered hexavalent Fc proteins with enhanced Fc-gamma receptor avidity provide insights into immune-complex interactions

**DOI:** 10.1038/s42003-018-0149-9

**Published:** 2018-09-14

**Authors:** Tania F. Rowley, Shirley J. Peters, Mike Aylott, Robert Griffin, Nicola L. Davies, Louise J. Healy, Rona M. Cutler, Alison Eddleston, Thomas L. Pither, Joshua M. Sopp, Oliver Zaccheo, Gianluca Fossati, Katharine Cain, Andrew M. Ventom, Hanna Hailu, Eleanor J. Ward, John Sherington, Frank R. Brennan, Farnaz Fallah-Arani, David P. Humphreys

**Affiliations:** 1 UCB Pharma, 216 Bath Road, Slough, SL1 3WE UK; 2 Statistical Consultant, St Albans, UK; 3 Aurelia Bioscience, Nottingham, UK; 4 Institute of Cellular Medicine, Newcastle University Medical School, Newcastle upon Tyne, UK; 5 Cancer Sciences Unit, Faculty of Medicine, University of Southampton, Southampton, UK; 6 Eurofins Pharma Bioanalysis Services UK Ltd, Abingdon, UK; 7 Vertex, Abingdon, UK; 8 Biochemical Pharmacology William Harvey Research Institute, Queen Mary University of London, London, UK

## Abstract

Autoantibody-mediated diseases are currently treated with intravenous immunoglobulin, which is thought to act in part via blockade of Fc gamma receptors, thereby inhibiting autoantibody effector functions and subsequent pathology. We aimed to develop recombinant molecules with enhanced Fc receptor avidity and thus increased potency over intravenous immunoglobulin. Here we describe the molecular engineering of human Fc hexamers and explore their therapeutic and safety profiles. We show Fc hexamers were more potent than IVIG in phagocytosis blockade and disease models. However, in human whole-blood safety assays incubation with IgG1 isotype Fc hexamers resulted in cytokine release, platelet and complement activation, whereas the IgG4 version did not. We used a statistically designed mutagenesis approach to identify the key Fc residues involved in these processes. Cytokine release was found to be dependent on neutrophil FcγRIIIb interactions with L234 and A327 in the Fc. Therefore, Fc hexamers provide unique insights into Fc receptor biology.

## Abstract (web summary)

Tania Rowley et al. present multivalent Fc molecules with enhanced avidity for Fc gamma receptors in order to improve the treatment of autoantibody-mediated human diseases. They found several key amino acids involved in Fc receptor binding interactions.

## Introduction

Immune effector cells respond to antibodies via the family of Fc gamma receptors (FcγR). In humans this family consists of six members: FcγRI (CD64), FcγRIIa/b/c (CD32a/b/c), and FcγRIIIa/b (CD16a/b). Excepting FcγRI, Fcγ receptors have low-affinity interactions with monovalent IgG. These receptors (and their allotypic variants) have characteristic preferences for the binding of IgG isotypes 1–4 (ref. ^1^) and exhibit differential cellular expression on leukocytes^2–4^. Furthermore, they can transduce both activating (e.g. FcγRI, IIa, IIIa) and inhibitory (e.g. FcγRIIb) signals. The complex interactions of FcγRs with IgGs controls a variety of immune effector functions such as antibody-dependent cellular phagocytosis by macrophages, antibody-dependent cellular cytotoxicity by natural killer cells, neutrophil degranulation, and platelet activation which are critical for immunity. However, the pathogenesis of many immune disorders is now associated with the aberrant production of autoantibodies with the consequent generation of damaging immune complexes.

Intravenous immunoglobulin (IVIG) is a pooled human immunoglobulin used to treat a number of immune conditions, including immune thrombocytic purpura (ITP), Guillain-Barré syndrome, and chronic idiopathic demyelinating polyneuropathy. These conditions involve pathogenic autoantibodies along with tissue destruction by FcγR-bearing effector cells^5,6^. One prospective mode of action of IVIG is the blockade of FcγRs by low levels of non-monomeric Ig present before infusion and formed by anti-idiotype networks after infusion^7^. IVIG products for human use must have >90% monomer/dimer and <0.5% aggregate, with the proportion of dimer ranging between 3% and 16% dependent on formulation^8,9^. Although dimers are largely reversible^10^ they have been proposed to be pharmacologically active and responsible for some of the infusion-related adverse events^9,11–13^. The FcγR blockade hypothesis is supported by humans trials where the Fc fragment, but not the F(ab′)_2_ fragment, was effective in treating ITP^14^. Furthermore, the phagocytic clearance of antibody-coated cells is slowed after IVIG infusion to ITP patients^15^. Additional support for specific FcγR-dependent mechanisms comes from the use of anti-Rhesus D sera to treat ITP patients^16^. Aggregate-rich or cross-linked IgG also appear to be more potent at blocking an experimental model of ITP in mice^17,18^.

We and others have postulated that high avidity multivalent Fc constructs might have potential for the treatment of immune disorders involving autoantibodies^19–23^. Such molecules might achieve effective receptor blockade at considerably lower doses than IVIG. However, this approach requires careful consideration of potential safety concerns. In particular, the induction of potentially serious acute events such as cytokine release, platelet activation/aggregation, and complement activation need to be carefully assessed. However, study of the IgG–FcγR axis faces translational science challenges. Mice have four receptors (FcγRI, RIIb, RIII, and RIV) with substantial differences from humans^24^, including lack of FcγRIIa on platelets and being prone to an IgE-like IgG-driven anaphylactoid response^25^. Primate FcγR receptors are a closer match to human in sequence, and in that their platelets express FcγRIIa. However, cynomolgus macaque granulocytes do not express FcγRIIIb but express compensating high levels of FcγRIIa^26,27^. Hence, in order to fully study FcγR biology relevant to human therapy, human in vitro studies must be used in combination with rodent and primate in vivo studies.

Here we describe the engineering of human IgG1 and IgG4 Fc domains into hexameric forms by fusion of the human IgM tailpiece to the Fc C-terminus^22,23,28–30^. Comparison of IgG1 vs IgG4 Fc hexamers in FcγR-blockade potency and safety assays showed strongly polarized effects. In order to maximize the potency-safety balance, we have dissected the involvement of the divergent IgG1/4 Fc CH2 domain residues using a mutagenesis strategy powered by Design of Experiments (DoE) statistical design. We have identified the key and subsidiary amino acids involved in IgG1-induced cytokine release, platelet activation and C1q binding and the receptor interactions they control. Furthermore, we show that substitution of the IgG4 versions of these amino acids allows the fine-tuning of FcγR binding, leading to altered cellular responses.

## Results

### Generation of Fc hexamers

**Fig. 1:**
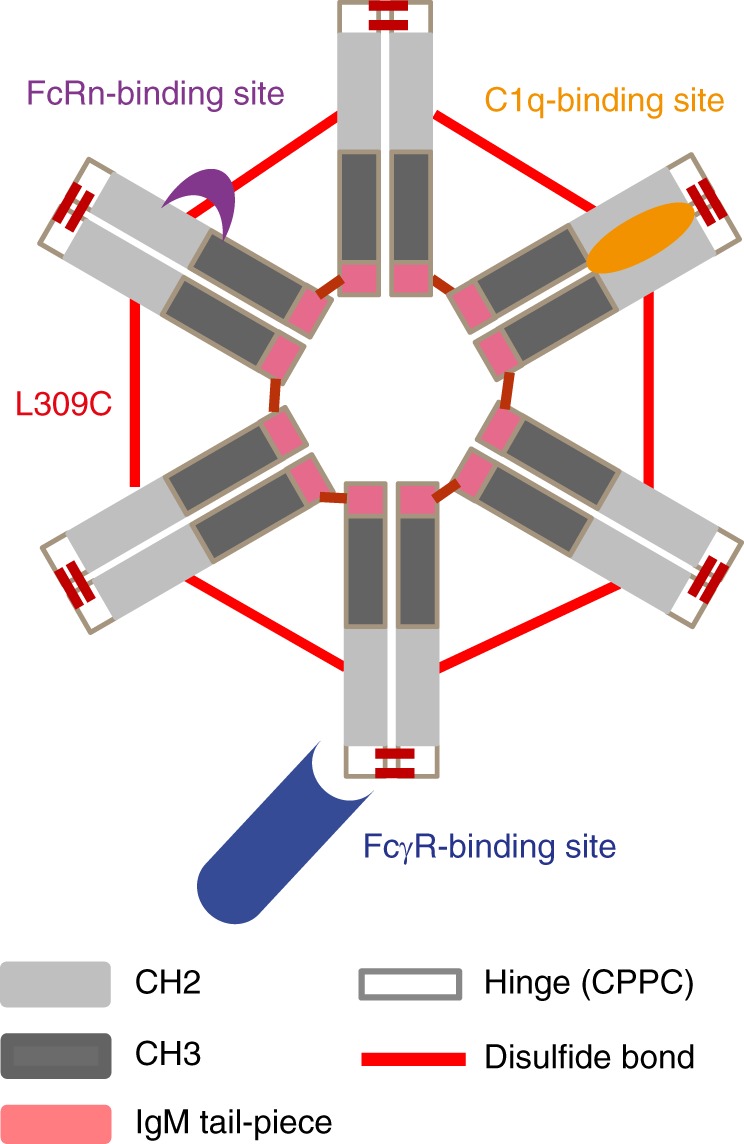
Schematic showing the structure of Fc hexamers. Fc hexamers consist of the human IgG Fc hinge-CH2-CH3 domains fused to an IgM tailpiece domain, which provides stable hexamerization. Fc-γ receptor (blue cylindar), C1q (orange oval), and FcRn (purple crescent)-binding sites and L309C mutation generating an additional cysteine bond (red) are indicated. Adapted from Qureshi et al.^22^

Human IgG1 and IgG4 Fc with mature N-termini starting with an IgG1 core hinge (CPPC) were each directly fused at their C-terminal lysine’s to the 18 amino acid C-terminal extension or tailpiece (PTLYNVSLVMSDTAGTCY) of human IgM (Fig. 1). Maintaining a native sequence is important to avoid potential immunogenicity risks and as such the wild-type hexamer has a 100% human sequence, lacking mutations or insertions^30,31^ (Supplementary Fig. 1). The dipeptide junction between Cγ3 and the IgM tailpiece is identical to that between Cμ4 and its tailpiece (Supplementary Fig. 2). The tailpiece contains a target motif for N-linked glycosylation and a penultimate cysteine known to be involved in IgM^575^ and IgA^471^ polymerization^32,33^. We observed hexamer in the presence or absence of L309C, which mimics the constant domain cysteine found in IgM^414^ and IgA^309^. Human IgG1 and IgG4 Fc tailpiece fused constructs were expressed in a transient CHO system and we observed good levels of expression from all constructs (450 ± 150 mg/l). Protein A-purified hexamer were typically 70–80% and 30–40% hexamer for IgG1 and IgG4 constructs, respectively, and >95% hexamer for both IgG1 and IgG4 constructs on analytical HPLC after size SEC purification (as detailed in Supplementary Fiqure 1 of Qureshi et al.^22^). Multiple bands were observed for the L309 construct on SDS-PAGE while a single band was observed for L309C (Supplementary Fig. 3).

### Fc hexamers block antibody-mediated phagocytosis by human macrophages in vitro and inhibit in vivo mouse models of ITP

**Fig. 2:**
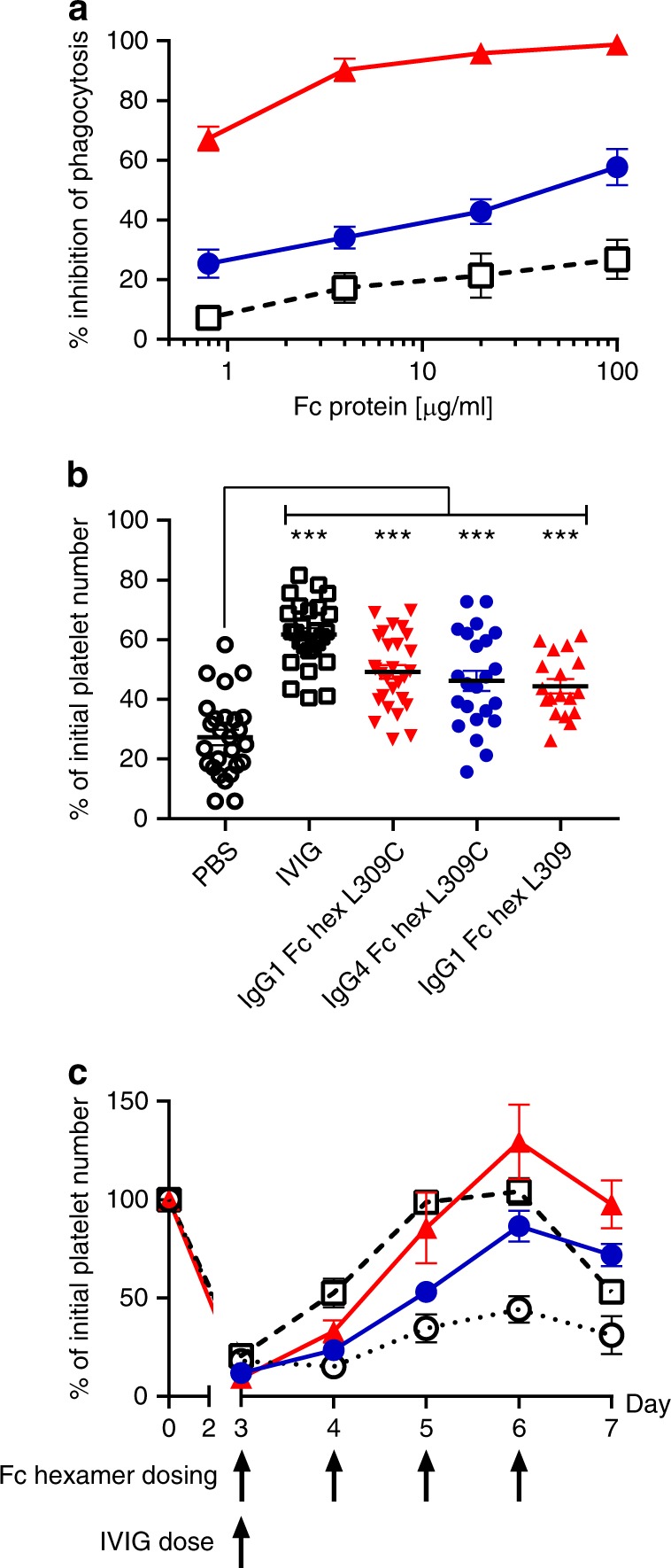
Fc hexamers block antibody-mediated phagocytosis by human macrophages in vitro and inhibit in vivo mouse models of ITP. **a** Inhibition of phagocytosis. Human macrophages were co-cultured with anti-CD20 opsonized target B cells and IgG1 (red triangles), IgG4 (blue circles) Fc hexamers (L309) or IVIG (open black boxes) were added at the indicated concentrations. Disappearance of target cells was measured by FACS after 18 h and % inhibition of phagocytosis calculated. Data are mean of *n* = 5 individual donors ± SEM. **b** Acute ITP model. ITP was induced by intraperitoneal administration of anti-CD41. IVIG (1000 mg/kg) or Fc hexamers (10 mg/kg) hexamers were administered i.v. 1 h prior to anti-CD41. Data are expressed as % of baseline platelet numbers with mean ± SEM and is a compilation of five individual experiments, *n* = 18–28/group. Statistical analysis was carried out by two-way ANOVA (experiments and treatment) and Dunnetts multiple comparison test (***p < 0.01) vs PBS controls. **c** Chronic ITP model. ITP was induced by subcutaneous implantation of an osmotic minipump containing anti-CD41. IgG1 (red triangles) or IgG4 (blue circles) Fc hexamer (L309C) was dosed at 10 mg/kg and IVIG (open black boxes) at 1000 mg/kg i.v. on the indicated day/s. Data are expressed as mean ± SEM of the % of baseline platelet numbers remaining, *n* = 6/group. Statistical analysis was carried out separately for days 4–7 by one-way ANOVA and Dunnetts multiple comparison test. The increase in platelet numbers for IgG1 Fc hexamer vs PBS controls (open black circles) was significant on days 5–7 (n.s. day 4, *p* < 0.01 day 5, *p* < 0.0001 days 6 and 7). The increase in platelet numbers for IgG4 Fc hexamer vs PBS controls was significant on days 6 and 7 (n.s. days 4 and 5, *p* < 0.05 day 6, *p* < 0.01 day 7). The increase in platelet numbers for IVIG vs PBS controls was significant on days 4–6 (*p* < 0.001 days 4 and 5, *p* < 0.01 day 6, n.s. day 7)

In order to recapitulate one of the proposed modes of action of IVIG, a phagocytosis blockade assay using human primary monocyte-derived macrophages was devised. Macrophage phagocytosis was chosen as the most functionally relevant assay since it involves dynamic interactions of multiple FcγR and autoimmune tissue destruction triggered by pathogenic IgG can be directly linked with macrophages^34^ and is blocked by Fc components within IVIG^15,17^. In vitro macrophage-colony-stimulating factor (MCSF)-differentiated macrophages were found to highly express FcγRI, II, and III and efficiently phagocytosed autologous B cell targets coated with an anti-CD20 IgG1 in an FcγR-dependent manner (Supplementary Fig. 4a, b). This phagocytosis process was dependent on FcγRI, II, and III, as demonstrated by the requirement for neutralizing antibodies against all three receptors for full blockade (Supplementary Fig. 4b). IgG1 Fc hexamer was able to fully block this process (99% at 100 μg/ml) and was highly potent compared to IVIG. IgG4 Fc hexamer was less potent than IgG1 but consistently showed enhanced activity over IVIG at all concentrations tested (58% block compared to 27% block by IVIG at 100 μg/ml) (Fig. 2a). IgG1 Fc hexamers ± L309C had identical activities in blockade of macrophage phagocytosis (Supplementary Fig. 4c).

We performed acute and chronic mouse models of ITP in wild-type mice to investigate the efficacy of Fc hexamers in vivo. In these models bolus administration (acute) or continuous infusion (chronic) of a rat IgG1 anti-mouse CD41 (Integrin α2b) causes platelet clearance in BALB/c mice. Previous work has shown that this antibody clears platelets via a predominantly mouse FcγRIII mechanism^35^ and this clearance can be inhibited by FcγR-blocking agents or large doses (~1000 mg/kg) of IVIG^17–19^. In the acute model, IgG1 and IgG4 Fc hexamers (with or without the L309C mutation) administered prophylactically before the anti-CD41 mAb reduced platelet depletion at a dose of 10 mg/kg (*p* < 0.01 vs phosphate-buffered saline (PBS) by two-way analysis of variance (ANOVA) and Dunnetts multiple comparison test). IVIG was also effective, but at a much larger dose of 1000 mg/kg (Fig. 2b). In the chronic ITP model, daily therapeutic dosing of 10 mg/kg IgG1 Fc hexamer after anti-CD41-induced platelet loss was as effective as a 1000 mg/kg single dose of IVIG at restoring platelet numbers. A statistically significant difference in platelet numbers compared to controls was seen on day 6 post ITP induction following treatment with either IVIG, IgG1, or IgG4 Fc hexamer (IVIG vs PBS *p* < 0.01; IgG1 Fc hexamer vs PBS *p* < 0.0001; IgG4 Fc hexamer vs PBS *p* < 0.05 at day 6 by one-way ANOVA and Dunnetts multiple comparison test), although the IgG1 Fc hexamer was more effective than the IgG4 Fc hexamer (Fig. 2c). Hence, both IgG1 and IgG4 Fc hexamers are effective FcγR-blocking agents in vitro and in vivo but with IgG1 Fc hexamer being more potent than IgG4.

Mice received repeat doses of Fc hexamers as our previous published data have demonstrated that both L309 and L309C Fc hexamers have a short circulating serum half-life, presumably as a result of high target-mediated clearance. However, this is balanced by extended pharmacology due to Fc hexamers triggering FcγR internalization^22^. We selected the native L309 variant for further engineering both to minimize immunogenicity risk and because L309 is adjacent to the H310 critical for FcRn binding. Fc hexamers containing the L309C disulfide bond have been shown by others to be capable of binding to FcRn at pH 6 (ref. ^23^), but our data suggest that the L309 variant may be more effective at interacting with FcRn than the L309C hexameric variant (Fig. 11 of patent WO15132364 (ref. ^36^)).

The in vivo activity of IVIG has been ascribed in some reports to the presence of α2,6 sialic acid and interaction with inhibitory receptors such as DC-SIGN, SIGN-R1, or FcγRIIb^6,37^. Our Fc hexamers were expressed in CHO cells, which are incapable of synthesizing α2,6 sialic acid due to lack of a functional α2,6-sialyltransferase I (ST6)^38,39^. Hence, modes of action via α2,6 sialic acid can be ruled out. Glycan analysis of the CH2 and tailpiece in Supplementary Table 1 showed no substantial α2,3 sialic acid. The CH2 glycans were typical of IgG and the tailpiece has approximately ~50% glycan occupancy with high mannose (Man5 and Man6) being the most abundant.

### Assessment of the potential toxicity risks of IgG1 and IgG4 Fc hexamers

**Fig. 3:**
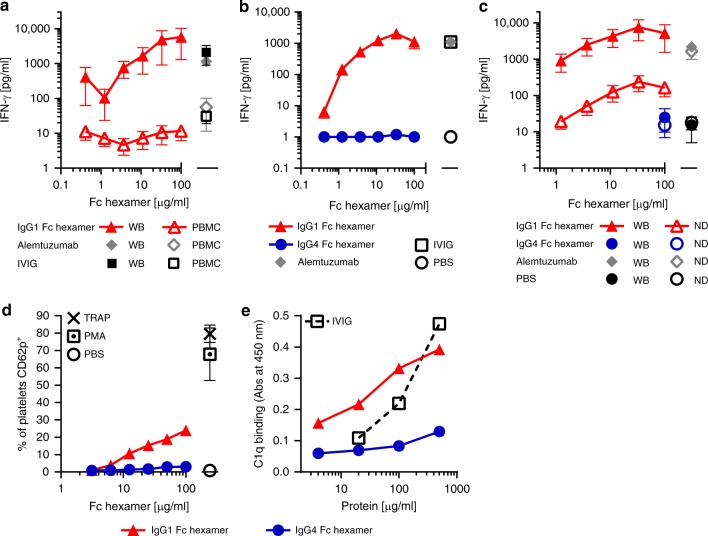
Assessment of the potential toxicity risks of IgG1 and IgG4 Fc hexamers. **a** Human whole blood vs PBMC cytokine release by Fc hexamers. Whole blood or PBMC from the same donors was cultured for 24 h with a titration of IgG1 Fc hexamer (L309C) or positive controls alemtuzumab (100 μg/ml) and IVIG (2232 μg/ml). Data represent mean ± SEM of plasma IFN-γ concentration, *n* = 3 donors. **b** Comparison of IgG1 and IgG4 Fc hexamers (L309) in whole-blood cytokine release. Blood was cultured for 24 h with a titration of Fc hexamers or controls. Data represent mean ± SEM of plasma IFN-γ concentration, *n* = 3 donors. **c** Depletion of neutrophils from whole blood reduces cytokine release. Whole blood (WB) or neutrophil-depleted (ND) blood was stimulated with a titration of IgG1 Fc hexamer or controls for 24 h. Data represent mean ± SEM of plasma IFN-γ concentration, *n* = 3 donors. **d** Comparison of IgG1 and IgG4 Fc hexamers in platelet activation. Human whole blood was incubated with a titration of Fc hexamers (L309) or positive controls (TRAP/PMA) at room temperature for 25 min. Data represent the mean ± SEM of % of platelets CD62p^+^, *n* = 3 donors. **e** Comparison of IgG1 and IgG4 Fc hexamers (L309) in C1q binding. Relative ability of Fc hexamers to bind C1q was measured by ELISA. Data represent mean ± SD of duplicate tests

Binding of high-avidity Fc hexamers has the potential to inadvertently induce inflammatory FcγR signaling by cross-linking receptors, leading to cytokine release^40^. We compared two cytokine release assay formats, human peripheral blood mononuclear cells (PBMC) and minimally diluted whole blood^41^, to test for cytokine release by Fc hexamers. IgG1 Fc hexamers did not trigger significant release of cytokines from isolated PBMCs. In contrast, the whole-blood assay demonstrated a very substantial release of cytokines such as interferon-γ (IFN-γ), tumor necrosis factor-α (TNF-α), interleukin-6 (IL-6), IL-8, and IL-1β plus low levels of IL-13, but no IL-2, IL-4, IL-10, or IL-12p70 in response to IgG1 Fc hexamers (Fig. 3a and Supplementary Fig. 6). The concentrations of cytokines released were similar to that observed with alemtuzumab or IVIG controls, which are known to cause cytokine release in the clinic^42,43^ (Fig. 3a, b). The high cytokine release by IVIG was surprising, but may be explained by the relatively high protein concentration (2232 μg/ml) used in the assay and the fact that the IVIG used had been buffer exchanged to remove excipients. This resulted in an increase in the proportion on non-monomeric species from ~5 to ~8% (Supplementary Fig. 7). Thus, the cytokine release seen in our assay may not be fully reflective of clinical grade IVIG administered to humans. No cytokine release was observed in the whole-blood assay after stimulation with IgG4 Fc hexamers (Fig. 3b and Supplementary Fig. 6). Hence, whole-blood assays, with a full complement of FcγR-bearing cells, are the more appropriate and sensitive assay format for detecting FcγR-driven cytokine release.

To further understand why the IgG1 Fc hexamers induced cytokine release from human whole blood, but not PBMCs, we performed cell subtraction studies to identify the cell types involved. As neutrophils are the predominant FcγR-bearing cell type lost during PBMC isolation, we specifically removed them from whole blood using magnetic anti-CD15 beads in order to leave other major blood components intact (Supplementary Fig 8). Neutrophil removal considerably reduced IFN-γ release by IgG1 Fc hexamers but had a more limited effect on alemtuzumab, which is known to involve NK cell mechanisms^42^ (Fig. 3c). These data suggest that neutrophils are a key component of the cytokine release cascade induced by Fc multimers. We investigated whether isolated human neutrophils are capable of secreting cytokines. Data (Supplementary Fig. 9) show that both ±L309C forms of IgG1 Fc hexamer produced IFN-γ and TNF-α while IgG4 Fc hexamer and a null form of IgG1 (LALA, L234A L235A) produced negligible levels. These data support a mechanistic link between hexameric Fc–FcγR interactions and IFNγ release. However, the concentration of cytokine produced by purified neutrophils does not match that seen in whole-blood assays, perhaps suggestive of the importance of neutrophil density or a network of cell stimuli in blood.

Another critical safety consideration for hexameric Fc is the possibility of platelet activation through engagement of FcγRIIa. Activation, typically witnessed by increased levels of CD62p (p-selectin) or release of serotonin, can reach a threshold where platelet aggregation occurs. Aggregation can seed microthrombi, which can result in life-threatening tissue thrombosis such as deep vein thrombosis, stroke, or heart attack. We utilized a human platelet activation assay measuring expression of CD62p as a marker of α-granule release and activation^44^. The data in Fig. 3d show that the IgG1 Fc hexamers caused a pronounced increase in the percentage of CD62p^+^ platelets. This platelet activation was donor-variable, ranging from ~15% to 60% across experiments. However, IgG4 Fc hexamer caused very low levels of platelet activation, typically ~2–5%.

Finally, interaction of Fc hexamers with the complement system was also considered. C1q binding by the Fc hexamers was compared using a C1q-binding enzyme-linked immunosorbent assay (ELISA). As shown in Fig. 3e, IgG1 Fc hexamers showed a clear ability to bind C1q with enhanced potency compared to IVIG at low concentrations. In contrast, IgG4 Fc hexamers had very low binding capacity for C1q, below that of IVIG, which is in line with the published inability of IgG4 antibodies to initiate complement activation^45^.

### Amino acid positions controlling cellular responses to IgG1 and IgG4 Fc hexamers identified using statistically designed mutagenesis

We wanted to understand the striking potency and safety differences between IgG1 and IgG4 Fc hexamers and explore whether differential FcγR binding was involved. IgG1 and IgG4 Fc have only 13 amino acid differences outside of the core hinge: 7 in the FcγR-contacting CH2 domain and 6 in the FcγR-distant CH3 (Supplementary Fig. 10). There are 2^7^ (or 128) potential combinations of the natural IgG1 vs IgG4 CH2 domain variants which were impractical to study in their entirety. Instead, we employed DoE statistical design to explore this sequence space. This allowed us to test the role of all variants using a panel of only 16 IgG1/4 combination mutants (Supplementary Table 2). In addition to efficiently identifying key individual amino acids, DoE with this design space has the power to identify interactions between co-dependent residues which may be important for subtle control of protein–protein binding interfaces.

**Fig. 4:**
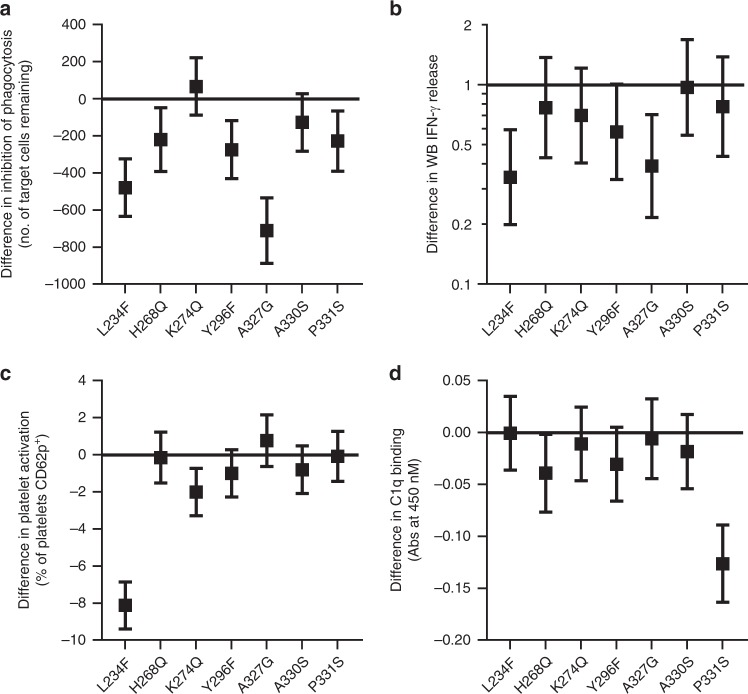
Amino acid positions that control the divergent cellular responses to IgG1 and IgG4 Fc hexamers can be identified using statistically designed mutagenesis. The DoE panel of 16 Fc hexamers were tested in efficacy (phagocytosis) and safety (WB cytokine release, platelet activation, C1q binding) assays. Results are displayed as effects plots including all seven IgG1/4 divergent CH2 residues. Data represent the difference in arithmetic means for graphs **a**, **c** and **d ** and the ratio of geometric means for graph **b** as compared to IgG1 WT. Error bars represent 95% confidence intervals. **a** Analysis of phagocytosis inhibition was performed using the mean target cell remaining values for DoE Fc hexamers at 0.8 and 4 μg/ml. *n* = 1 donor. **b** Analysis of WB cytokine release was performed using the geometric means of cytokine release (IFN-γ, pg/ml) for DoE Fc hexamers at 33 μg/ml. A log-transformation (base 10) was applied to the data before analysis. *n* = 3 donors. **c** Analysis of platelet activation was performed using the mean % of platelets CD62p^+^. The overall mean across donors was taken for DoE Fc hexamers at 100, 50, and 25 μg/ml. *n* = 3 donors. **d** Analysis of C1q binding was performed on the mean of Abs 450 nm values for DoE Fc hexamers at 500 and 100 µg/ml

All 16 DoE constructs were purified as hexamers and assessed in in vitro efficacy (human macrophage phagocytosis blockade) and safety (whole-blood cytokine release, platelet activation, and C1q binding) assays. The results are summarized as a series of effect plots in Fig. 4a–d where the differences in the mean response for each residue are compared to IgG1 wild-type hexamers. Any amino acid position which separates significantly from baseline is indicated to be involved in the assayed activity. It is important to note that effects plots show the involvement of particular single amino acids even though they had been tested in the context of multiple other mutations. For blockade of FcγR-mediated phagocytosis, analysis (Fig. 4a) showed that the A327G (IgG1 (A) to IgG4 (G)) mutation had the biggest effect on reducing potency in blockade of phagocytosis (*p* = 0.00003), with L234F also having a major inhibitory effect (*p* = 0.00016) and H268Q, Y296F, and P331S additionally having weak but significant inhibitory effects (H268Q *p* = 0.01896, Y296F *p* = 0.0042, P331S *p* = 0.01275). An interaction was observed between L234F and P331S, with P331S in its IgG4 (S) setting reducing the ability of the Fc hexamer to block phagocytosis when combined with L234F in its IgG4 (F) setting (Supplementary Fig. 11a). Regarding whole-blood cytokine release, analysis showed that only L234F and A327G mutations significantly reduced cytokine release (L234F *p* = 0.00199, A327G *p* = 0.00648) and there were no observed interactions (Fig. 4b). L234F also had a dominant inhibitory effect on platelet activation (*p* = 0.000004) with K274Q having a statistically significant (*p* = 0.00814) but minor role (Fig. 4c). There was also evidence for statistical interaction between L234F and K274Q, with K274Q having a more noticeable effect when L234F was in its IgG4 (F) setting (Supplementary Fig. 11c). Hence, it appears that L234 and K274 might be central to FcγRIIa binding on platelets by Fc hexamers. There was also a minor interaction between L234F and A330S (Supplementary Fig. 11b). For C1q binding, P331S had the dominant inhibitory effect (*p* = 0.00005) and H268Q had a minor inhibitory effect (*p* = 0.04223) (Fig. 4d). This observation is in keeping with the known role of the P331 residue in C1q binding by IgG1 antibodies^45^.

### Confirmation of the amino acid positions controlling the divergent cellular responses to IgG1 and IgG4 Fc using single mutants

**Fig. 5:**
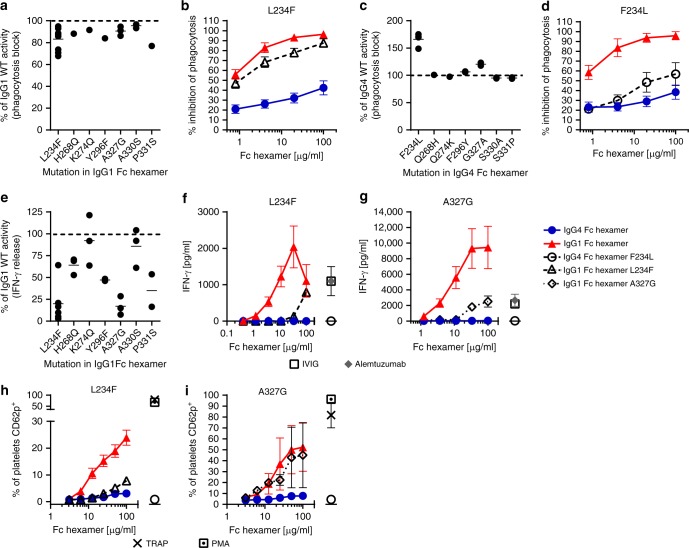
Confirmation of amino acid positions that control the divergent cellular responses to IgG1 and IgG4 Fc hexamers using single mutants. **a** Inhibition of phagocytosis by IgG1 Fc hexamer single mutants (20 μg/ml). % of IgG1 activity was calculated as (mutant % block/IgG1 WT % block in same experiment) × 100%. Data represent the individual donor response and mean of *n* = 1–9 donors. **b** Inhibition of phagocytosis by IgG1 Fc hexamer L234F. Data represent the mean ± SEM of *n* = 9 donors. **c** Inhibition of phagocytosis by IgG4 Fc hexamer single mutants (20 μg/ml). % of IgG4 activity was calculated as (mutant % block/IgG4 WT % block in same experiment) × 100%. Data represent individual donor response and mean of *n* = 1–4 donors. **d** Inhibition of phagocytosis by IgG4 Fc hexamer F234L. Data represent the mean ± SEM of *n* = 4 donors. **e** IgG1 Fc hexamer single mutant (50/33.3 μg/ml) activity in WB cytokine release. % of IgG1 IFN-γ release was calculated as (mutant IFN-γ release/IgG1 WT IFN-γ release in same experiment) × 100%. Data represent individual donor response and mean of *n* = 2–6 donors. **f** Whole-blood cytokine release by IgG1 Fc hexamer L234F. Data represent the mean ± SEM of *n* = 3 donors. **g** Whole-blood cytokine release by IgG1 Fc hexamer A327G. Data represent the mean ± SEM of *n* = 3 donors. **h** Platelet activation by IgG1 Fc hexamer L234F. Data represent the mean ± SEM of *n* = 3 donors. **i** Platelet activation by IgG1 Fc hexamer A327G. Data represent the mean ± SD of *n* *=* 2 donors

To confirm involvement of the identified residues, we made all seven single CH2 mutations on both wild-type IgG1 or IgG4 background sequences and tested them in the phagocytosis blockade assay. In the IgG1 hexamer background, all of the mutations alone caused only marginal decreases in the magnitude of FcγR blockade (Fig. 5a, b). However, in the context of the IgG4 hexamer background, the F234L mutation showed improved blockade of FcγR-mediated phagocytosis (Fig. 5c, d), indicating the pivotal role of the L234 residue for binding to FcγRs involved in phagocytosis. Whole-blood cytokine release results showed that the L234F and A327G mutations on the IgG1 background had the biggest impact in reducing release of IFN-γ (Fig. 5e–g), confirming the role of L234 and A327 in cytokine release processes. None of the seven single mutations in the IgG4 Fc background induced IFN-γ beyond wild-type IgG4 Fc hexamer levels (cytokine release was equivent to background PBS-only values or zero for all mutants). Taken together, these data suggest that either L234F or A327G could improve the safety of IgG1 Fc hexamer by reducing cytokine release with limited impact on FcγR blockade efficacy. The converse mutation F234L has the potential to improve the efficacy of IgG4 Fc hexamers without impacting cytokine-related safety. In keeping with our DoE observations, the L234F but not the A327G mutation on IgG1 was capable of potently reducing platelet activation (Fig. 5h, i). Hence, the L234F mutation in IgG1 Fc offered the potential for both reducing cytokine release and platelet activation but without major loss of efficacy in the phagocytosis blockade assay.

**Fig. 6:**
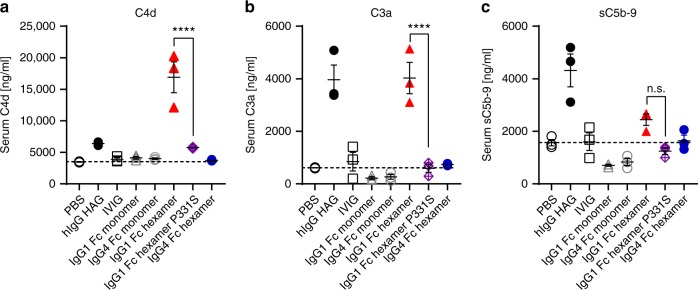
Induction of the complement cascade by IgG1 Fc hexamer is ablated by the P331S mutation. Fluid-phase C4d (**a**), C3a (**b**), and sC5b-9 (**c**) were measured in human serum after 1 h incubation with the indicated human IgG Fc constructs (100 μg/ml) or heat-aggregated IgG (HAG) controls. Graphs show the individual donor data (*n* *=* 3) with mean ± SEM. Statistical analysis was carried out by two-way ANOVA (donors and stimulation) and Tukey’s multiple comparison test. PBS vs IgG1 Fc hexamer stimulation *p* < 0.0001 for C4d and C3a, n.s. for sC5b-9. IgG1 Fc hexamer vs P331S mutant *p* < 0.0001 for C4d and C3a, n.s. for sC5b-9

In order to examine the ability of Fc hexamers to trigger the complement cascade further than C1q binding, we tested them in a human serum assay measuring production of C4d, C3a, and sC5b-9 (Fig. 6). IgG1 Fc hexamers strongly induced C4d and C3a (*p* < 0.0001, two-way ANOVA) but only weakly stimulated sC5b-9, as compared to heat-aggregated IgG. IgG4 Fc hexamers did not meaningfully stimulate any of the measured complement components. This suggested that there was an initial activation of the complement cascade by IgG1 Fc hexamers that did not fully progress to terminal complement complex (sC5b-9) formation. The P331S mutation significantly reduced the production of C4d and C3a (*p* < 0.0001, two-way ANOVA) in the serum assay to background levels (Fig. 6).

### L234F and A327G in the IgG1/4 Fc CH2 domain control cellular responses to Fc hexamers by altering their affinity for specific Fcγ receptors

**Fig. 7:**
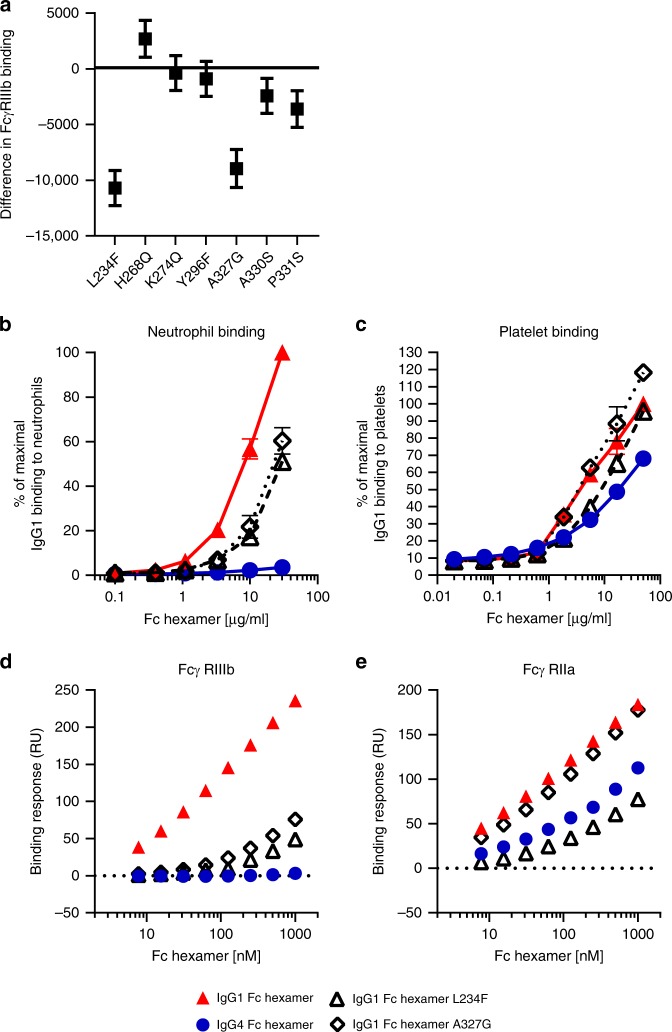
L234F and A327G in the IgG1/4 Fc CH2 domain control cellular responses to Fc hexamers via altering their affinity for specific FcγR. **a** DoE analysis of FcγRIIIb binding. Binding of Alexafluor-647-labeled DoE Fc hexamers to HEK cells expressing FcγRIIIb was measured by flow cytometry. DoE analysis was performed using the mean binding at 3, 1, and 0.3 μg/ml Fc hexamer. Results are displayed as an effects plot with data representing the difference in arithmetic mean from IgG1 WT. Error bars represent 95% confidence intervals. **b** Single mutant IgG1 Fc hexamer L234F and A327G binding to neutrophils in whole blood. Data represent the mean ± SD of *n* *=* 2 donors. **c** Single mutant IgG1 Fc hexamer L234F and A327G binding to platelets. Data represent the mean ± SEM of *n* *=* 3 donors heterozygous for R/H131 FcγRIIa polymorphism. **d**, **e** Surface plasmon resonance analysis of L234F and A327G IgG1 Fc hexamer single mutants. Equilibrium binding response of Fc hexamers binding to **d** FcγRIIIb and **e** FcγRIIa

We considered whether these residues were fine tuning the interaction of Fc hexamers with the different FcγR expressed on the involved cell types. Initial comparison of the DoE panel of 16 IgG1/4 mutants binding to HEK cells transfected with the main allotypes of each of the six human Fcγ receptors indicated the only receptor showing major variance of binding was FcγRIIIb. Statistical analysis identified L234F and A327G as the most significant residue changes which reduce IgG1 Fc hexamer binding to FcγRIIIb (L234F *p* = 8.48E-07, A327G *p* = 5.01E-06), as shown in Fig. 7a. The only cell type known to express FcγRIIIb is neutrophils and furthermore we had previously shown they are critical players in whole-blood cytokine release. Thus, we hypothesized that the difference observed in these mutants on cytokine release vs platelet activation may be due to L234F and A327G both affecting binding to neutrophils (expressing FcγRIIa and IIIb) vs L234F being the only one of these two affecting binding to platelets (expressing only FcγRIIa).

Non-physiological, high expression of single FcγR on transfected HEK cells may mask subtle changes in Fc affinity. We therefore tested binding of single mutant Fc hexamers to human neutrophils and platelets in whole blood. Both L234F and A327G mutants demonstrated reduced binding to human neutrophils in whole blood compared to IgG1 wild-type hexamers (A327G 60% and L234F 51% of wild-type binding, Fig. 7b). In contrast, binding to platelets was reduced by the L234F mutant but unaffected by A327G (Fig. 7c) in line with the observed effect on platelet activation. This suggested that A327G reduces IgG1 Fc affinity for FcγRIIIb, whereas L234F reduces affinity for both FcγRIIIb and FcγRIIa. This supposition was confirmed by testing IgG1 Fc hexamer single mutants L234F and A327G for their binding to FcγRIIIb and FcγRIIa by surface plasmon resonance. The BIAcore response plots for FcγRIIIb and FcγRIIa (Fig. 7d, e, sensorgrams shown in Supplementary Fig. 12) show L234F and A327G both reduced Fc binding to FcγRIIIb to a similar extent but only the L234F mutation also reduced Fc binding to FcγRIIa.

## Discussion

This study demonstrates that Fc hexamers are very effective at FcγR blockade and as such potently inhibit FcγR-mediated phagocytosis of antibody-targeted cells in vitro and in vivo in murine models of ITP^19,46^. We further demonstrate that the subtle changes in Fc sequence which occur between IgG1 and IgG4 isotypes result in dramatic differences in safety assay readouts as high valency Fc molecules. We have used DoE statistical methods to engineer hybrid Fc hexamers with altered characteristics. Use of this method has led to identification of the pivotal Fc CH2 residues involved in cytokine release (A327 and L234) and platelet activation (L234), and confirmed the pre-eminence of P331 in C1q binding and activation of the complement cascade.

The stable hexameric Fc format described here provided a platform for unique insight into Fc–FcγR interactions and Fc variant effects. Previous studies have commonly employed immune complexes of heterogeneous valency and structural complexity, such as the use of anti-hapten monoclonal antibodies^47^. Such reagents are excellent for study of the biology of soluble immune complexes, but are perhaps less applicable to the study of Fc interactions of antibodies against cell surface antigens, which are known to arrange hexamerically during cell killing^48^. Hence, Fc hexamers may have utility as an antibody Fc engineering tool. The influence on FcγR binding of single or compound mutants within monovalent Fc is difficult to study accurately using surface plasmon resonance because of low affinity. Hexamerization provides a robust method for increasing functional affinity (avidity), but also significantly increases the analytical challenge. Our own published data illustrate the complexity of analyzing Fc–FcγR binding interactions using hexameric Fc^22^. The functional avidity is influenced by Fc sequence, FcγR receptor sequence, allotype, density and putative dimerization, glycan heterogeneity, and the asymmetric nature of the Fc–FcγR interaction. At a molecular level the residues shown to be key in this study have been highlighted previously in structural and mutagenic studies^49,50^. However, this study moves beyond simple monovalent binding differences to complex functional consequences which can be witnessed only when subtle binding alterations have been compounded by avidity.

Cross-linking of FcγR using multivalent Fc has the potential to induce inflammatory signaling cascades, leading to cytokine release^40,51^. We show the fundamental importance of using human whole blood, not PBMC assays, to study multivalent Fc proteins and immune-complexes. The critical FcγR-dense neutrophils and platelets are missing from commonly used PBMC assays and as such we observed no cytokine release with IgG1 Fc hexamer in a human PBMC assay, but profound release in a minimally diluted whole-blood assay. Depletion of neutrophils from whole blood suggests that they are key mediators of this cytokine response in human blood. Isolated neutrophils have been shown to be capable of producing IFN-γ here and previously^52,53^. The critical disparity between IgG1 (high cytokine release) and IgG4 (no cytokine release) Fc hexamers may be linked to their differential ability to bind FcγR on neutrophils in whole blood. Neutrophils express FcγRIIa and high levels of FcγRIIIb at rest and can induce the expression of FcγRI upon activation^54^. It has been reported by others that IgG4 immune complexes are not capable of binding to FcγRIIIb effectively, in contrast to IgG1 (ref. ^1^). This suggests FcγRIIIb binding on neutrophils may be at the heart of the differential cytokine release by the IgG1 Fc hexamers compared to IgG4. This observation is consistent with recent reports showing that FcγRIIIb is involved in neutrophil activation processes^55–57^ and NETosis^58,59^, and not simply a decoy receptor.

In our experience, mice were not useful models for studying cytokine-mediated toxicities by human IgG Fc hexamers. Wild-type mice showed signs of a temporary anaphylactoid-type response, with transient hunched posture and pilo-erection after intravenous (i.v.) administration of Fc hexamers. However, both IgG1 and IgG4 Fc hexamers appeared to affect mice equally, in marked contrast to the in vitro human whole-blood response. Furthermore, the cytokines released by mice did not match those seen in human whole blood. There was no measurable IFN-γ or TNF-α, but evidence of IL-6, KC, and IL-10 lasting 1–2 h in BALB/c mice^22^. We found the anaphylactoid response to be strain dependent; BALB/c mice had the mildest and shortest response (lasting for ~1 h) with C57BL/6 mice transgenic for human FcγR^60^ having a more severe response. The effects on FcγR transgenic mice made functional and safety studies of Fc hexamers in these animals unworkable, even after pre-administration of anti-histamines and PAF inhibitors. Hence, rodents did not replicate all relevant aspects of human FcγR biology, focusing our studies on human whole-blood assays.

DoE statistical design is widely used in bioprocessing and other sciences, but has rarely been applied to protein engineering. Here, we have used this technique to effectively identify residues which control distinct Fc effector functions and receptor interactions. Namely, in IgG1 Fc L234 and A327 both contribute to cytokine release, L234 additionally controls platelet activation and P331 is critical for C1q binding and activation of the complement cascade. Furthermore, this DoE analysis has demonstrated that contributions to FcγR binding can be multi-factorial, as evidenced by statistical secondary interactions such as K274 (to primary L234) in platelet activation (i.e. FcγRIIa binding) and P331 (to primary L234) in inhibition of macrophage phagocytosis. We also show the secondary involvement of H268 and Y296 in inhibition of macrophage phagocytosis where these residues had not previously been implicated in FcγR binding. The involvement of L234 in FcγR binding is known in the LALA, FALA, LE, and LV null mutants^61–66^ and has been studied as L234F but only in the context of multiple additional mutations^67,68^. A327 has also been implicated in FcγRII/III binding in the context of additional null mutants^69,70^. L234 and A327 are contact residues between IgG1 and FcγRIIa^49^. However, our data show that the IgG4 versions of these residues can be minimally substituted alone while profoundly modulating effector responses. Our data also provide further insights into how these residues control primary cell responses, such as cytokine release in whole blood, which involves a myriad of potential interactions. This has revealed a rather surprising subtlety in discrimination between extracellular domains of receptors with different biological roles. IgG1 with either L234F or A327G both have reduced cytokine production (neutrophil-driven, expressing FcγRIIa and FcγRIIIb). However, L234F but not A327G substitutions reduced platelet activation (express FcγRIIa). The binding data shown here suggest that A327G and L234F both equally reduce affinity for FcγRIIIb, but only L234F additionally reduces FcγRIIa binding. This suggest that L234F and A327G mutations reduced cytokine release through altered neutrophil FcγRIIIb triggering, indicating a previously unappreciated role for this receptor in Fc-mediated cytokine release.

The IgG1 Fc hexamers appeared capable of triggering the complement cascade in serum, at least up until the point of C3a release. This observation is broadly in line with Spirig et al.^23^, who have shown Fc hexamers may deplete complement by partial activation of the cascade which does not lead to terminal complex sC5b-9 deposition. We found that the native P331S mutation could ablate serum complement activation by Fc hexamers, while only having a very limited effect on their ability to interact with Fc receptors and block macrophages phagocytosis, thus demonstrating the ability this approach has to fine-tune molecular interactions according to functional requirements.

In conclusion, we have characterized multivalent Fc variants which may have future potential in the clinic as recombinant IVIG mimetics for treatment of autoantibody-mediated diseases. Our extensive mutagenesis and in vitro human cell assays have highlighted that Fc isotype is a key aspect involved in potential toxicities with these molecules. We show that use of statistically powered Fc engineering along with human in vitro functional assays can be a powerful approach for identifying mutants which specifically modulate cellular responses, even when they are controlled by multiple receptors. The translatability of these human in vitro assays to safety in animal models is currently being investigated. However, given the complexity of human FcγR biology and imperfect match to mice and primates, clinically relevant translational studies for multivalent Fc remain a challenge. Nonetheless, we demonstrate the power of harnessing evolutionarily selected isotype sequence variations to adjust desired Fc domain effector functions.

## Methods

### Construct design, expression, and purification

Fc multimers were expressed and purified as described previously in Qureshi et al.^22^. DNA constructs as described in Results were ordered from DNA 2.0 (now called ATUM) and further mutations were introduced by mutagenesis using Quikchange Lightning Site-Directed Mutagenesis (Agilent). Protein sequences are shown in Supplementary Fig. 1 and oligonucleotide primers used are listed in Supplementary Table 3. Constructs were transfected into CHO cells using proprietary electroporation^71^. CHO cells were diluted to 2 × 10^8^ cells/ml in cold Earls balanced Salts solution. For each cuvette to be electroporated, 800 μl cells were mixed with 400 μg DNA. Each cuvette was subjected to the following parameters: 1 ms at 9.6 A, 10 ms at 0 A, and 40 ms at 3.2 A. After electroporation, the contents of three cuvettes were transferred to 250 ml ProCHO5 media (supplemented with 2 mM Glutamax) and incubated overnight at 37 °C, 5% CO_2_ on a platform shaking at 140 RPM. On day 1 post-transfection the incubator temperature was reduced to 32 °C and cells were cultured for a further 13 days before harvest. Supernatant was harvested by centrifugation at 40,000 RPM for at least 45 min followed by filtration through a 0.22 μm stericup filter. Hexamers were purified using a MabSelect sure Protein A affinity chromatography column (GE Healthcare), followed by S200 size exclusion chromatography to purify out the hexamer fraction. Endotoxin levels were low, typically <0.1 EU/mg. Hexamers were stored at 4 °C in PBS or frozen in aliquots at −80 °C.

### Human primary blood and cell assays

UK ethical approval for the use of NHSBT blood cones was obtained from NRES Committee South Central—Oxford C. Blood samples obtained from healthy controls at UCB Celltech were taken with informed consent under UCB Celltech UK HTA license number 12504.

### Human macrophage antibody-dependent phagocytosis

Phagocytosis assays were performed as described previously in Qureshi et al.^22^. Human PBMC were isolated from NHSBT blood cones by density-gradient centrifugation and monocytes selected by adherence. Monocytes were differentiated into macrophages by 7-day culture in 100 ng/ml MCSF (R&D). Autologous B cells were prepared from stored (frozen at −80 °C in 10% DMSO, 90% fetal bovine serum) non-adherent PBMCs by negative selection using MACS (B cell isolation kit II; Miltenyi Biotech) and labeled with CFSE (Molecular Probes). Differentiated macrophages and B cells were co-cultured at a 5:1 ratio in the presence of 0.1 μg/ml anti-CD20 (Rituximab, Biogen Idec/Genentech) to induce antibody-dependent phagocytosis of the B cell targets. Fc hexamers or controls were added at the indicated concentrations and the cells incubated at 37 °C, 5% CO_2_, 100% humidity for 18 h before analysis of remaining CFSE-labeled B cell targets by flow cytometry. A BD FACSCANTO II was used for acquisition in conjunction with BD FACSDIVA software. FlowJoV9.4.3 software was used for analysis. B cells identified by first gating live cells on FSC vs SSC, followed by gating the CFSE (FITC) + SSC low-labeled B cell population. Total B cell numbers per well were calculated from this gate. % inhibition of phagocytosis was calculated as ((value−background)/(max−background)) × 100%, where value = remaining B cells in the presence of multimeric-Fc or control plus anti-CD20, Max = remaining B cells in the absence of anti-CD20, and background = remaining B cells in the presence of anti-CD20 alone.

### Whole-blood cytokine release assays

Blood from healthy human volunteers was collected into lithium heparin vacutainers (BD) and used within 2 h of the blood draw. In some experiments, neutrophils were depleted by addition of anti-CD15 coated magnetic beads (Miltenyi MACSiBeads) to whole blood for 15 min at room temperature with rotation. The blood was then exposed to a magnetic field and the supernatant aspirated as the neutrophil-depleted fraction. Cells before and after depletion were stained for surface expression of CD15 PerCp (Biolegend, 323018), CD3 BV421 (Biolegend, 300434), CD19 FITC (BD, 555412), CD14 APC (BD, 555399), and CD56 PE (BD, 555516) and analyzed by flow cytometry using PKH26 beads (Sigma) as a reference for normalization. Acquisition was done on a BD FACS Canto II and analysis was done on FlowJo v10. Remaining neutrophils in depleted blood were ≤0.11% of leukocytes in all donors (Supplementary Fig. 8). Unseparated whole blood or neutrophil-depleted blood was stimulated with either the indicated concentrations of Fc hexamer, 100 μg/ml alemtuzumab (MabCampath from Genzyme,) or 2232 μg/ml IVIG (Gamunex, buffer exchanged to PBS). Firstly, 12.5 μl of 20× final concentration Fc hexamer or control reagent was diluted in PBS and transferred to a 96-well round bottom tissue culture plate (Costar).  Secondly, 237.5 µl of whole blood or neutrophil-depleted whole blood was added and mixed gently. Plates were incubated at 37 °C, 5% CO_2_, 100% humidity for 24 h, centrifuged at 1800 rpm for 5 min, plasma collected, and cytokines measured by MSD multiplex (IFN-γ, TNF-α, IL-6, IL-8, IL-1β, IL-13, IL-2, IL-4, IL-10, IL-12p70, Mesoscale Discovery, N05049A-1) read and analyzed using a MSD Sector Imager 6000. Plasma not analyzed immediately was stored at −80 °C until analysis.

### Platelet activation

Five microliters of human whole blood was added to 100 μl of media (RPMI + 5% human AB serum) containing the indicated concentrations of Fc hexamer or positive controls. Thrombin Receptor Activator Peptide (TRAP; Sigma) was used at 10 μg/ml and Phorbol 12-myristate 13-acetate (PMA; Sigma) was used at 20 ng/ml. Samples were incubated at room temperature for 25 min in the presence of anti-CD42b-APC (BD, 551061) and anti-CD62p-PE (BD, 550561). Samples were then fixed in 1% formaldehyde (Thermo Fisher) diluted in PBS and analyzed by flow cytometry. Platelets identified by plotting SSC-A vs CD42b APC-A and gating SSC low, CD42b+ platelets. The % of gated platelets CD62p+ was then determined. BD FACSCANTO II was used for acquisition in conjunction with BD FACSDIVA software. FlowJoV9.4.3 was used for analysis.

### C1q binding

Relative ability to bind C1q was measured using a C1q Ab ELISA kit (Abnova) according to the manufacturer’s protocol.

### Fluid-phase complement activation

Complement activation in human serum was determined by measuring C3a, C4d, and sC5b-9 concentrations. Fc hexamers or controls (100 μg/ml) were incubated in normal human serum for 1 h at 37 °C. C3a, C4d, or sC5b-9 concentrations were measured in the serum by ELISA (MicroVue EIA Kits, Quidel) according to the manufacturer’s protocol.

### Murine ITP models

All animal experiments were carried out in accordance with the Animals (Scientific Procedures) Act 1986 and were approved by the UCB Animal Welfare and Ethical Review Body. Male BALB/c mice greater than 6 weeks of age (Charles River, UK) were used.

Acute ITP: Mice were dosed with either 10 mg/kg Fc hexamer, 1000 mg/kg IVIG or PBS control i.v. One hour later platelet loss was induced by the intraperitoneal administration of 1 µg/mouse anti-CD41 (MWReg30; EBioscience). Blood samples were taken immediately prior to Fc hexamer/IVIG dosing and 24 h post anti-CD41 administration to measure baseline and final platelet numbers. Statistical analysis was carried out by one-way ANOVA and Dunnetts multiple comparison test.

Chronic ITP: Platelet loss was induced by continuous subcutaneous infusion of anti-CD41. Mice were implanted with an Alzet subcutaneous osmotic minipump discharging anti-CD41 at 82.5 µg/ml at a flow rate of 0.5 µl/h. Blood samples were taken prior to minipump insertion and again 72, 96, 120, 144, and 168 h later and used to determine platelet number. IVIG was administered as a single dose of 1000 mg/kg, i.v. at 72 h post implantation of the minipump. Fc hexamers were dosed daily, at 10 mg/kg i.v., from 72 h post implantation. Control animals received PBS. Statistical analysis was carried out by one-way ANOVA and Dunnetts multiple comparison test.

### Measurement of platelet numbers in murine ITP models by flow cytometry

Platelet number was determined by flow cytometry staining with CD45 and CD42d. Briefly, 5 μl mouse whole blood was collected into a heparinized tube and was placed into 100 μl FACS stain (anti-CD45 PerCP.Cy5.5, Ebioscience 45-0451-82; anti-CD42d PE, Ebioscience 12-0421-82). Samples were stained 20 min at 4 °C then 5 ml FACS buffer (PBS; 0.1% BSA) was added to each sample. Fifty microliters of sample was transferred to a 96-well plate and 150 μl FACS buffer added to each sample well. BD FACSCANTO II was used for acquisition with BD FACSDIVA software. FlowJoV9.4.3 was used for analysis. Initial gating carried out on FSC/SSC as platelets are significantly smaller than other cell types. Gated cells then gated as CD45−ve; plotted as SSC (*y-*axis) against CD45 PerCP.Cy5.5 (*x-*axis). Gated cells then gated as CD42d+ve; plotted as SSC (*y-*axis) against CD42d PE (*x-*axis). Platelets were judged to be the CD45− CD42d+ population (see Supplementary Fig. 5 for gating strategy)

### Statistical methods

Hybrid IgG1/4 hexamers were constructed using DoE statistical methodology^72^. We created a 2^(7−3)^ fractional factorial design consisting of seven factors (amino acids) and 16 runs (hexamer sequences) (Supplementary Table 2), where the lower setting for each amino acid was classed as IgG1, and the upper setting classed as IgG4. This was considered appropriate as an initial screening study to uncover twin and triple compound mutations. Data were analyzed using a linear model for main effects of amino acids and estimable two-factor interactions. Two-factor interactions that were not statistically significant (*p* ≥ 0.05) were removed from the model using a backward elimination process. Main effects of all the seven amino acids were retained in the model, irrespective of statistical significance. The eliminated interaction terms were included in the residual variance for estimating standard errors and confidence intervals. The results for the main effects are summarized as a series of effects plots with 95% confidence intervals. The cytokine release data were log_10_ transformed prior to statistical analysis, with the factor effects back-transformed to give ratios of geometric means. Analysis was performed with Design Expert version 9 (DX9) statistics software from Sat-Ease; final figures were generated using GraphPad prism version 7.02 software.

### Surface plasmon resonance

Surface plasmon resonance binding analysis was performed using a Biacore T200 (GE Healthcare). In all, ≈3000 response units of Anti-Tetra His Antibody (Qiagen) were immobilized on a CM5 Sensor Chip via amine coupling chemistry. HBS-EP+ buffer (10 mM HEPES pH 7.4, 0.15 M NaCl, 3 mM EDTA, 0.05% Surfactant P20; GE Healthcare) was used as the running buffer. At the start of each cycle the Fcγ receptors (R&D Systems) were captured to a different flow cell at approximately 250 response units and the baseline was allowed to stabilize. Fc hexamers were titrated over each receptor at concentrations from 7.8 to 1000 nM using a 60 μl injection at 30 μl/min. The surface was regenerated by 2 × 10 μl injections of 50 mM HCl, separated by a 5 μl injection of 5 mM NaOH at a flow rate of 10 μl/min. Reference subtraction was performed using a blank flow cell and blank analyte injections. The binding response 5 s prior to the end of the analyte injection (following blank subtraction) was plotted against Fc hexamer concentration and presented using GraphPad Prism 6.

### Fluorescent labeling of Fc hexamers and binding by flow cytometry

Binding to FcγR-expressing HEK cells: Alexafluor-647-labeled Fc hexamers (Life Technologies; A20186) were diluted in FACS buffer (DPBS + 0.2% BSA + 0.1% NaN_3_) at the indicated concentrations and incubated for 30 min at 4 °C with HEK cells expressing the NA1 variant of FcγRIIIb (ATCC, transfected in-house). Binding to granulocytes: 25 μl of Alexafluor-647-labeled Fc hexamer constructs were incubated with 75 μl of human whole blood for 30 min at 4 °C. Red blood cells were lysed (ACK lysis buffer; Gibco, A1049201) and binding was analyzed by flow cytometry. Granulocytes were gated by their expression of CD15 (Biolegend, 323004). Binding to platelets: 100 μl of Alexafluor-647-labeled Fc hexamer constructs were incubated with 5 μl of human whole blood for 30 min at 4 °C. Red blood cells were lysed (ACK lysis buffer) and the samples fixed in 1% paraformaldehyde (Thermo Fisher) prior to analysis. Platelets were gated by side scatter profile. BD FACSCANTO II was used for acquisition with BD FACSDIVA software. FlowJoV9.4.3 was used for analysis.

## Electronic supplementary material

Supplementary Information

## Data Availability

The datasets generated during and/or analyzed during the current study are available from the corresponding author on reasonable request. Unique materials will be made available on reasonable request.
