# Supplementary material for: Engineered hexavalent Fc proteins with enhanced Fc-gamma receptor avidity provide insights into immune-complex interactions
Source: Commun Biol. 2018 Sep 14;1:146. doi: 10.1038/s42003-018-0149-9 (PMC6138732; doi:10.1038/s42003-018-0149-9)
Supplement: Supplementary file 1 — Supplementary Information [file 42003_2018_149_MOESM1_ESM.pdf]

Supplementary Information

Supplementary Figures

1. **Human IgG1 Fc hexamer IgM tailpiece**
   CPPCPAPELLGGPSVFLFPPKPKDTLMISRTPEVTCVVVDVSHEDPEVKFNWYVDGVEVHNAKTKPREE
   QYNSTYRVVSVLTVLHQDWLNGKEYKCKVSNKALPAPIEKTISKAKGQPREPQVYTLPPSRDELTKNQV
   SLTCLVKGFYPSDIAVEWESNGQPENNYKTTPPVLDSDGSFFLYSKLTVDKSRWQQGNVFSCSVMHEAL
   HNHYTQKSLSLSPGK<u>PTLYNVSLVMSDTAGTCY</u>

2. **Human IgG1 Fc hexamer IgM tailpiece L309C**
   CPPCPAPELLGGPSVFLFPPKPKDTLMISRTPEVTCVVVDVSHEDPEVKFNWYVDGVEVHNAKTKPREE
   QYNSTYRVVSVLTV**C**HQDWLNGKEYKCKVSNKALPAPIEKTISKAKGQPREPQVYTLPPSRDELTKNQV
   SLTCLVKGFYPSDIAVEWESNGQPENNYKTTPPVLDSDGSFFLYSKLTVDKSRWQQGNVFSCSVMHEAL
   HNHYTQKSLSLSPGK<u>PTLYNVSLVMSDTAGTCY</u>

3. **Human IgG4 Fc hexamer IgM tailpiece**
   CPPCPAPEFLGGPSVFLFPPKPKDTLMISRTPEVTCVVVDVSQEDPEVQFNWYVDGVEVHNAKTKPREE
   QFNSTYRVVSVLTVLHQDWLNGKEYKCKVSNKGLPSSIEKTISKAKGQPREPQVYTLPPSQEEMTKNQV
   SLTCLVKGFYPSDIAVEWESNGQPENNYKTTPPVLDSDGSFFLYSRLTVDKSRWQEGNVFSCSVMHEAL
   HNHYTQKSLSLSLGK<u>PTLYNVSLVMSDTAGTCY</u>

4. **Human IgG4 Fc hexamer IgM tailpiece L309C**
   CPPCPAPEFLGGPSVFLFPPKPKDTLMISRTPEVTCVVVDVSQEDPEVQFNWYVDGVEVHNAKTKPREE
   QFNSTYRVVSVLTV**C**HQDWLNGKEYKCKVSNKGLPSSIEKTISKAKGQPREPQVYTLPPSQEEMTKNQV
   SLTCLVKGFYPSDIAVEWESNGQPENNYKTTPPVLDSDGSFFLYSRLTVDKSRWQEGNVFSCSVMHEAL
   HNHYTQKSLSLSLGK<u>PTLYNVSLVMSDTAGTCY</u>

Supplementary Fig. 1. Protein sequence of human IgG1 and IgG4 Fc wild-type hexamer constructs. IgM tailpiece residues are underlined. The cysteine mutation at position 309 in the L309C variants is indicated in bold.

**IgG1 Fc hexamer**	QKSLSLSPGK/PTLYNVSLVMSDTAGTCY
**IgG4 Fc hexamer**	QKSLSLSLGK/PTLYNVSLVMSDTAGTCY
**IgM**	ERTVDKSTGK/PTLYNVSLVMSDTAGTCY

Supplementary Fig. 2. Protein sequence comparison of Fc hexamer and IgM C-termini. Identical residues are underlined.

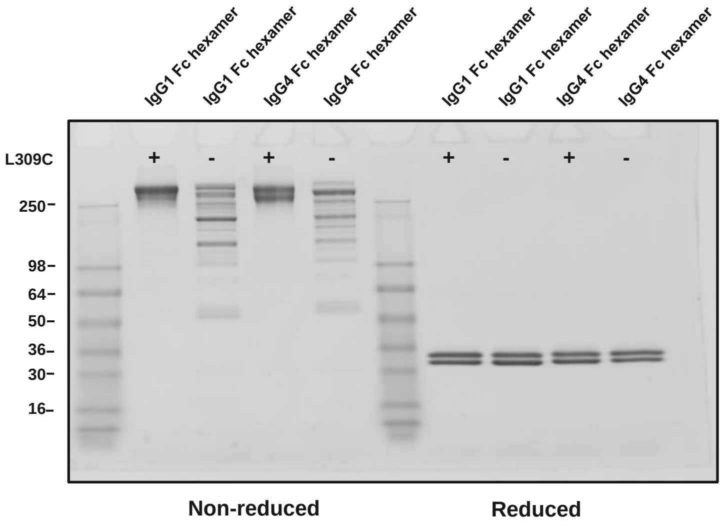

Supplementary Fig. 3. Analysis of purified Fc hexamers post gel filtration by SDS-PAGE. All samples were subjected to non-reducing (left) and reducing (right) conditions and resolved on a 4-20 % Tris-Glycine gel.

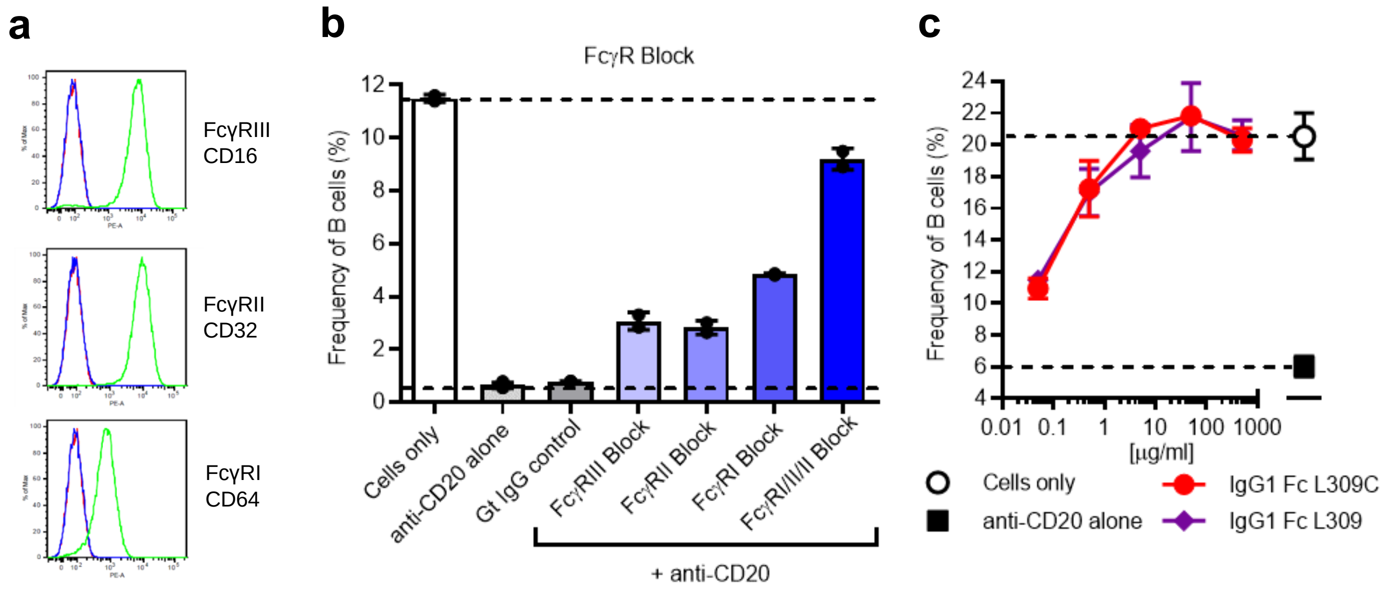

Supplementary Fig. 4. FcγR expression and dependence for macrophage phagocytosis. **a** Expression of FcγR I, II and III on human monocyte–derived macrophages. Macrophages were stained with anti-CD16/32/64 mAbs (BD) (green lines), isotype controls (blue) or unlabeled (red) and analyzed by flow cytometry. **b** FcγR-dependence of phagocytosis. Macrophage phagocytosis assay was set up as described in methods and FcγR blocked by addition of 33 μg/ml of individual blocking mAbs against FcγRI (R&D, AF1257) FcγRII (R&D, AF1330) or FcγRIII (Biolegend, 302033) or a combination. The % of target B cells remaining in the culture was determined after 18 h by flow cytometry. **c** L309 or L309C versions of IgG1 Fc hexamers display equal *in vitro* efficacy in blockade of phagocytosis. Macrophage phagocytosis assay was set up as described in methods with a titration of IgG1 Fc hexamers ± L309C mutation. The % of target B cells remaining in the culture was determined after 18 h by flow cytometry.

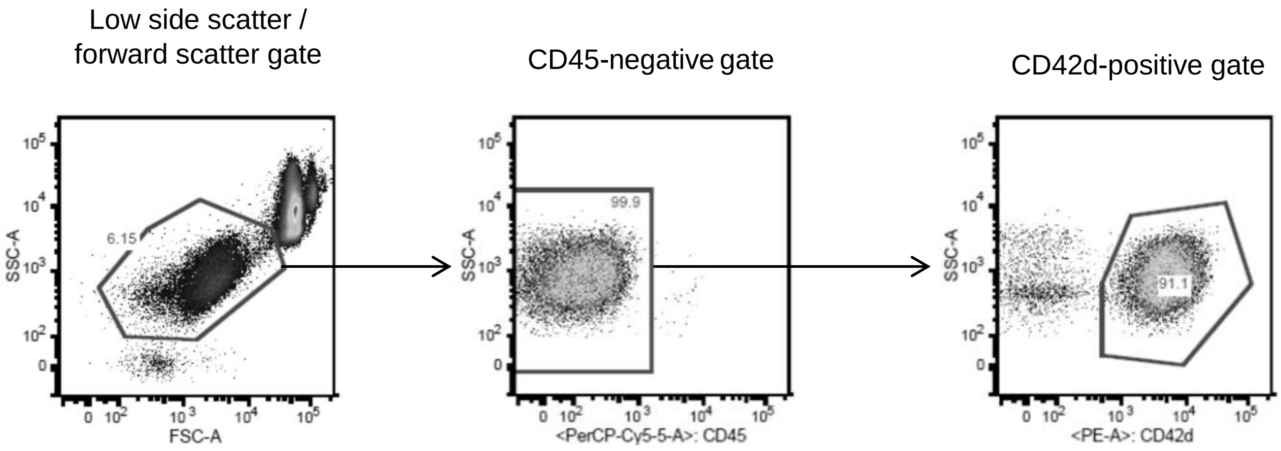

Supplementary Fig. 5. Flow cytometry gating strategy for identifying platelets in murine ITP model blood samples.

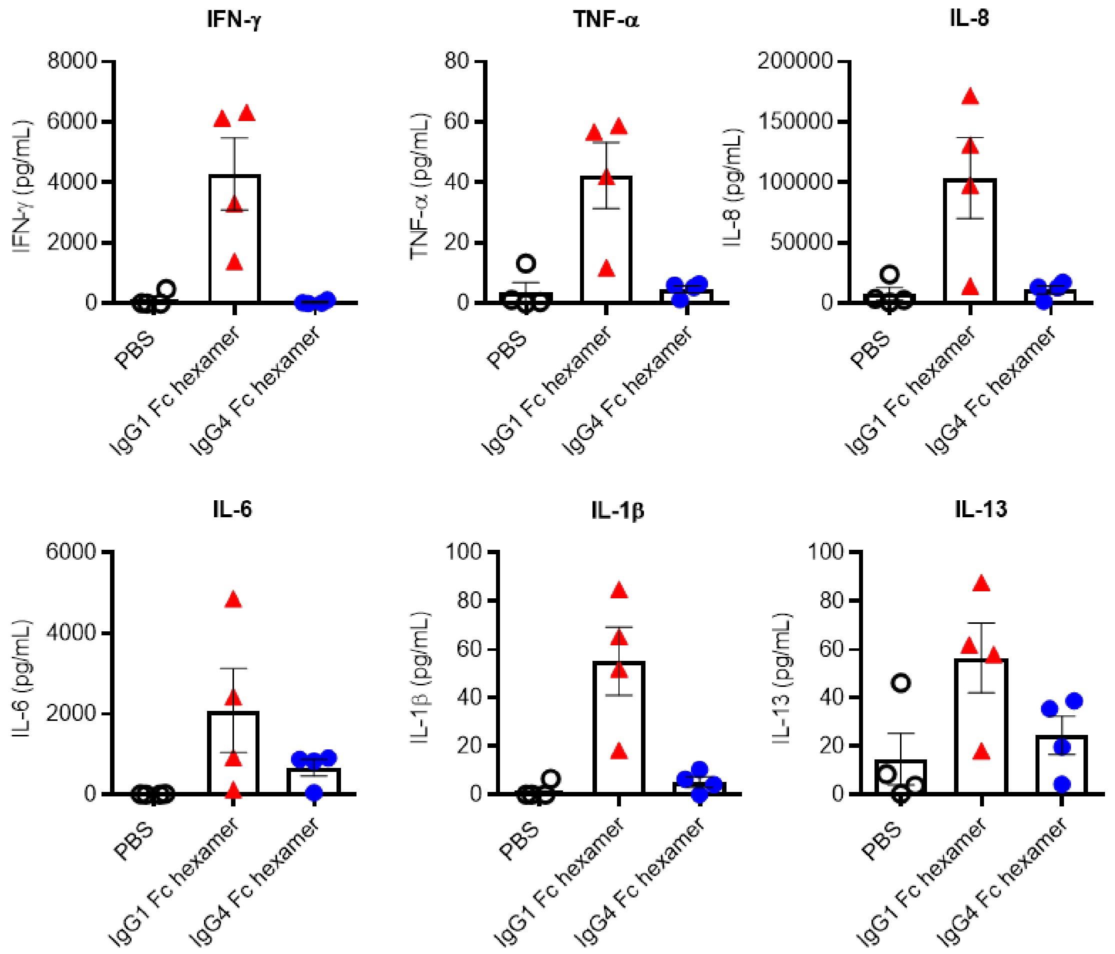

Supplementary Fig. 6. IgG1 Fc hexamers induce pro-inflammatory cytokine release. Human whole blood was cultured for 24 h with IgG1 or IgG4 Fc hexamers (L309C, 50 μg/ml). Plasma was analyzed for pro-inflammatory cytokines by MSD multiplex. No detection of IL-2, IL-4, IL-10 or IL-12p70 was observed over background controls. Data represent mean ± SEM of *n* = 4 donors.

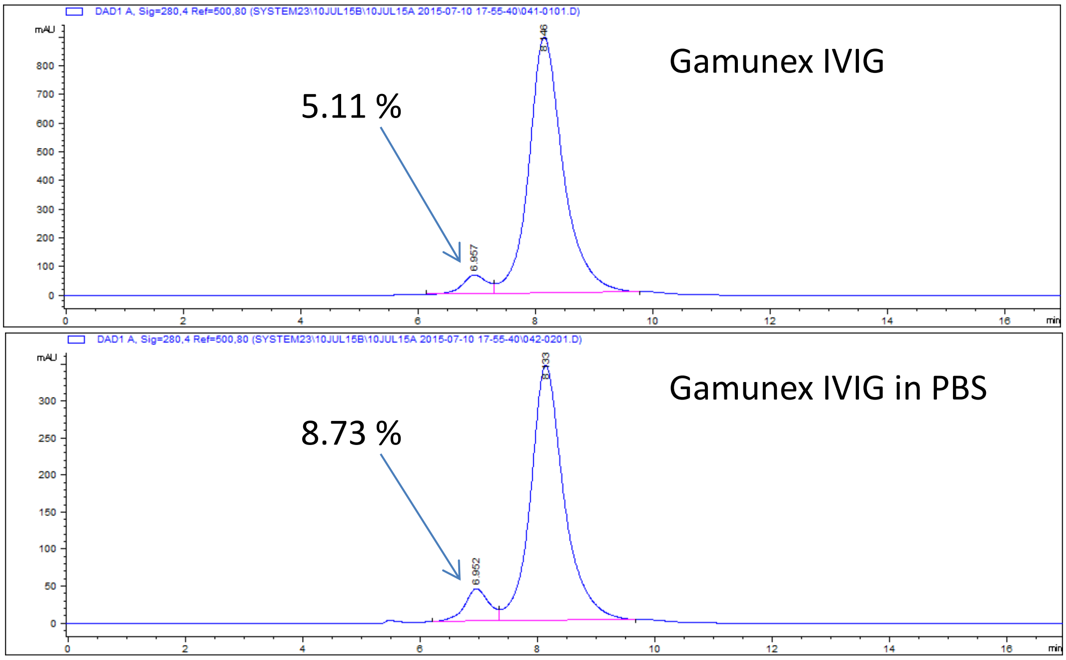

Supplementary Fig. 7. SEC analysis of IVIG before and after buffer exchange to PBS. Gamunex IVIG was buffer exchanged into PBS, pH 7.4 using a Centricon 70 concentrator before TSK-G3000 SE-HPLC analysis.

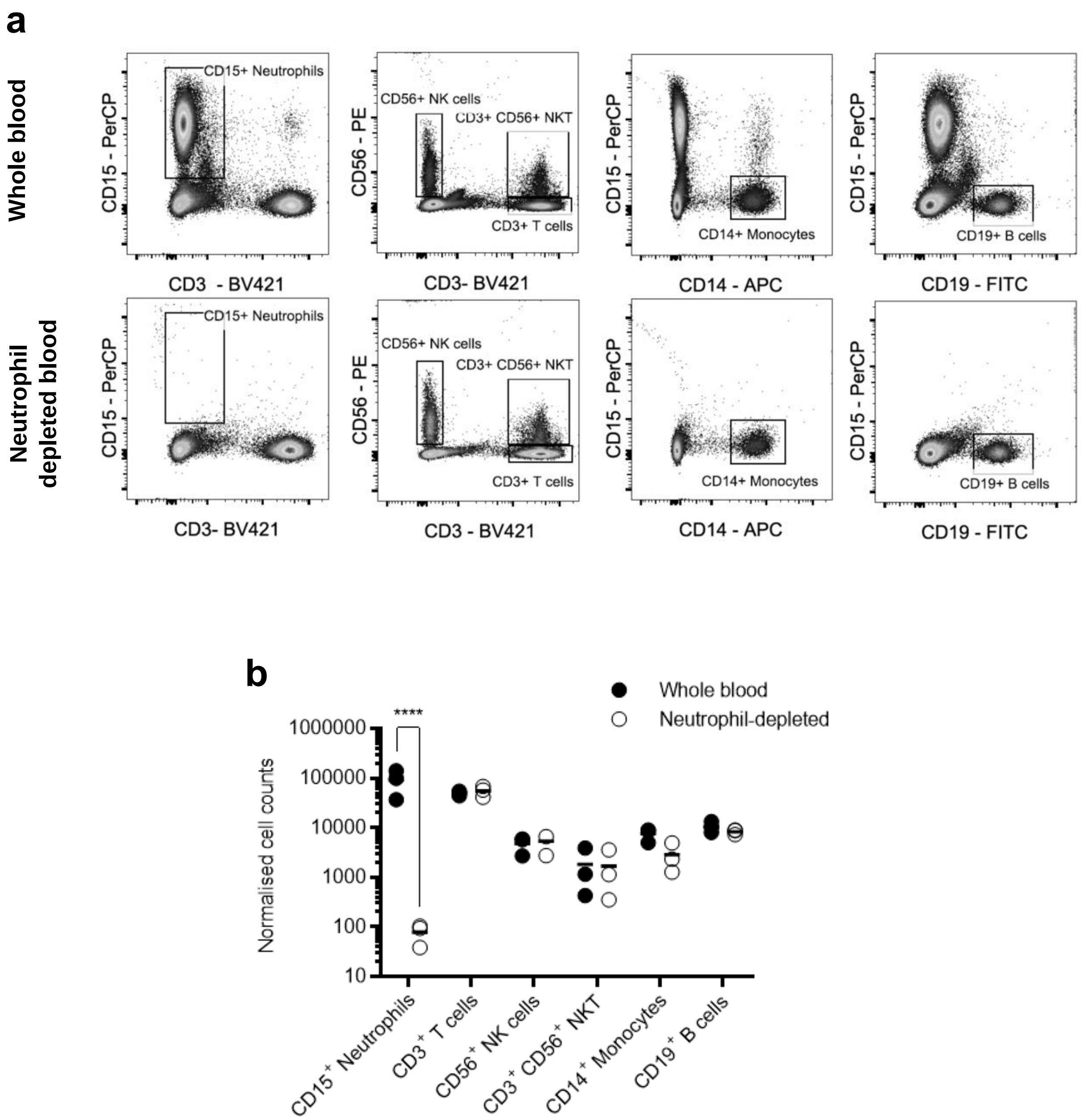

Supplementary Fig. 8. Cell subset analysis of neutrophil-depleted whole blood. **a** Example flow cytometry plots of whole and neutrophil-depleted blood from one representative donor out of three used in the experiment. **b** Normalized leukocyte subset counts from donors used for cytokine release assay before and after neutrophil depletion with anti-CD15 beads. Graphs show the individual donor data (n = 3) and mean of leukocyte counts normalized per 100,000 PKH26 reference beads collected. Statistical analysis of whole vs neutrophil depleted blood was done by 2-way Anova with Sidak's multiple comparisons test. $CD15^+$ Neutrophils were significantly reduced (p = <0.0001). Other cell subsets changes were not significant.

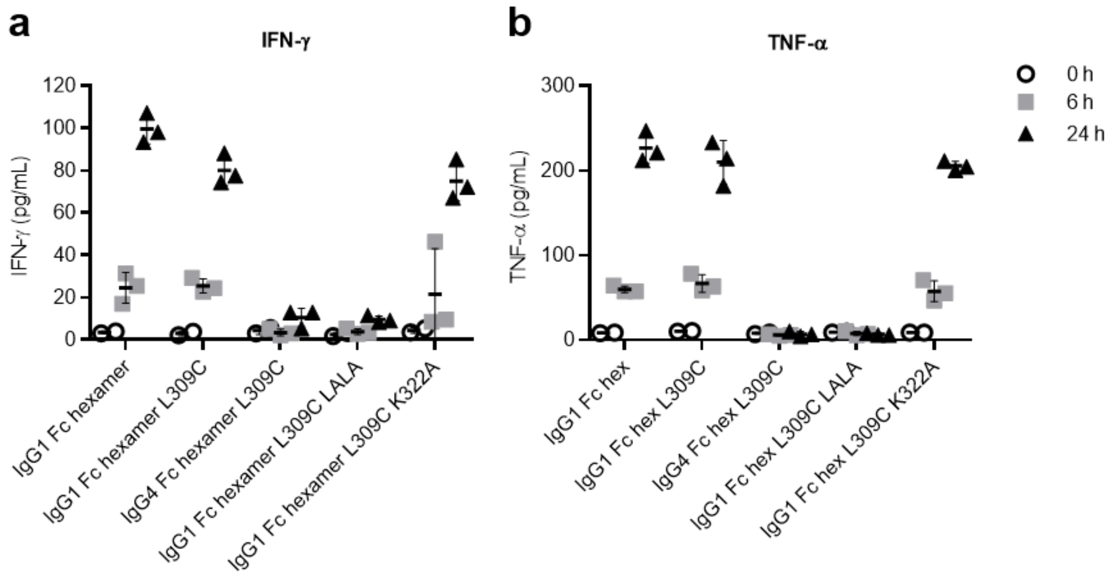

Supplementary Fig 9. Isolated neutrophils stimulated with Fc hexamers produce pro-inflammatory cytokines. Human neutrophils were isolated as described in the supplementary methods and were stimulated with the indicated Fc hexamers at 10 μg/ml for 0, 6 or 24 h. IFN-γ (**a**) or TNF-α (**b**) were measured in supernatants by MSD multiplex. Data represent the mean ± SD of duplicate or triplicate tests.

IgG1 (A)PE**L**$_{234}$LGGPSVFLFPPKPKDTLMISRTPEVTCVVVDVS**H**$_{268}$EDPEV**K**$_{274}$FNWYVD
GVEVHNAKTKPREEQ**Y**$_{296}$*NST*YRVVSVLTVLHQDWLNGKEYKCKVSNK**A**$_{327}$LP**A**$_{330}$**P**$_{331}$IEK
TISKAK
↑

IgG4 (A)PE**F**$_{234}$LGGPSVFLFPPKPKDTLMISRTPEVTCVVVDVS**Q**$_{268}$EDPEV**Q**$_{274}$FNWYVD
GVEVHNAKTKPREEQ**F**$_{296}$*NST*YRVVSVLTVLHQDWLNGKEYKCKVSNK**G**$_{327}$LP**S**$_{330}$**S**$_{331}$IEK
TISKAK
↑

Supplementary Fig. 10. Difference in the CH2 domain between human isotypes 1 and 4. Residues that differ are in bold and underlined. The glycosylation site is illustrated by italics and highlighted with an arrow. Numbering according to the EU numbering system.

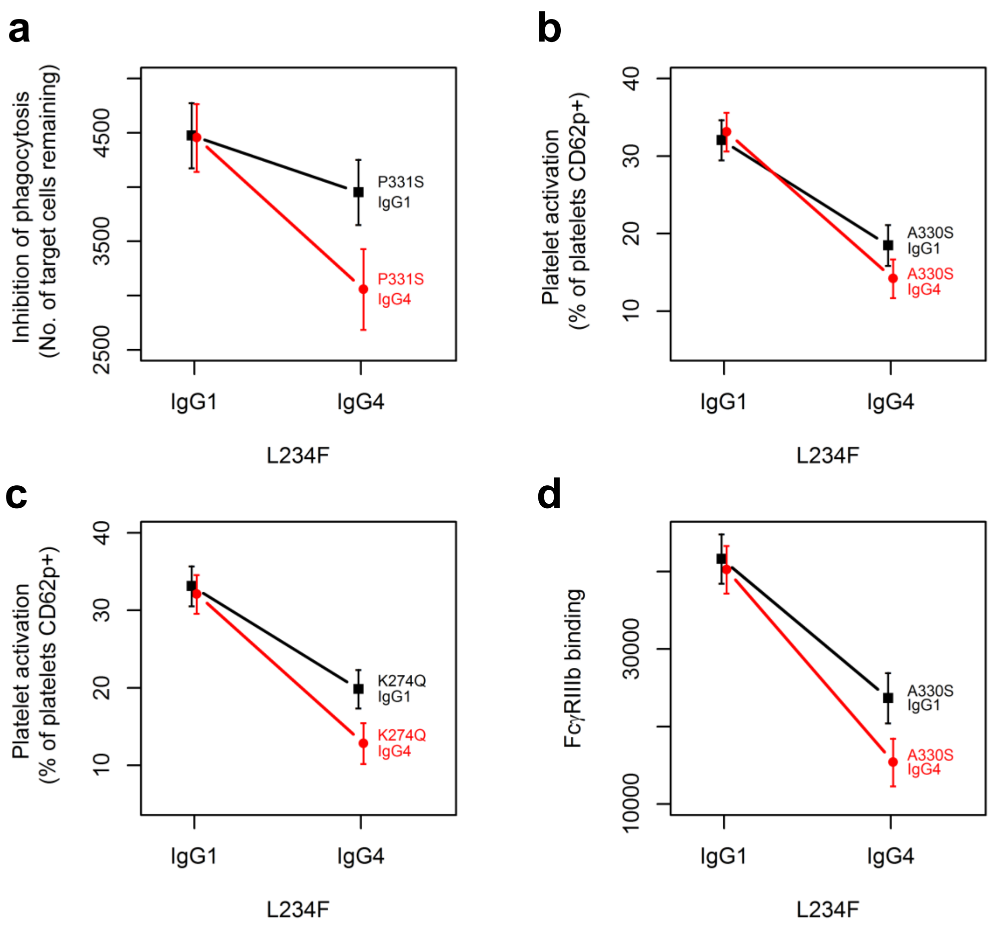

Supplementary Fig. 11. DoE interaction plots. Illustration of two-way interactions detected in the assays. **a** L234F by P331S interaction for phagocytosis inhibition. **b** L234F by A330S interaction for platelet activation. **c** L234F by K274Q interaction for platelet activation. **d** L234F by A330S interaction for FcγRIIIb binding.

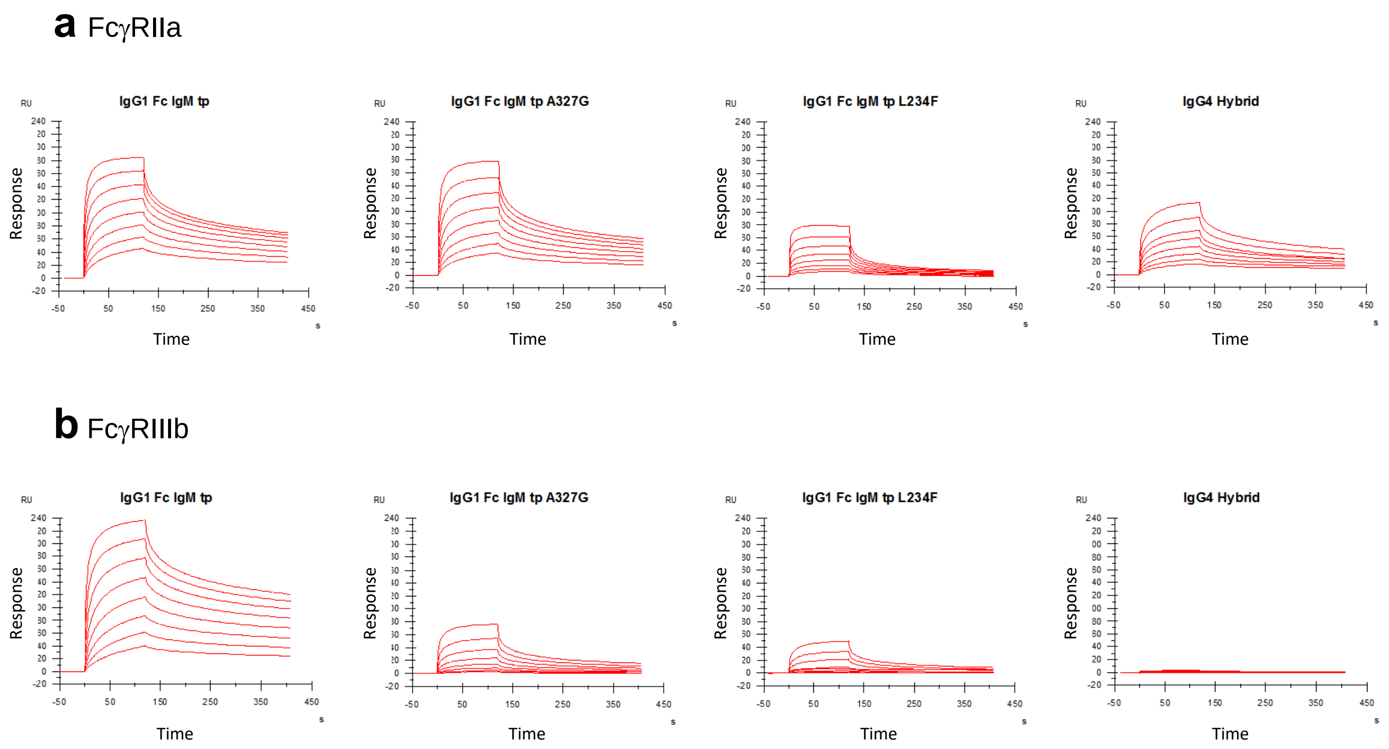

Supplementary Fig. 12. Sensorgrams showing the response observed when each Fc hexamer (as indicated) was titrated (at 1000, 500, 250, 125, 62.5, 31.3, 15.6 and 7.8 nM) over either captured FcγRIIa (**a**) or FcγRIIIb (**b**).

Supplementary Tables

	Site	Occupancy	G0F-N	G2-F	G1-F	G0-F	M6	M5
**IgG1 Fc IgM tailpiece L309C**	**CH2 N297**	93%	14%	5%	20%	38%	1%	15%
	**Tail piece N563**	59%	<1%	7%	5%	2%	24%	20%
**IgG1 Fc IgM tailpiece**	**CH2 N297**	91%	14%	<1%	26%	34%	<1%	15%
	**Tail piece N563**	45%	1%	4%	3%	<1%	14%	22%

Supplementary Table 1. Fc hexamer glycan analysis. Analysis was performed as indicated in supplementary methods.

Glycan structure names

G0F-N	Fucosylated pentasaccharide complex-type core N-glycan with single antennary N-acetylglucosamine residue
G2-F	Asialo- core-fucosylated bi-antennary complex-type N-glycan with two terminal galactose residues
G1-F	Asialo- core-fucosylated bi-antennary complex-type N-glycan with one terminal galactose residue
G0-F	Asialo- core-fucosylated bi-antennary complex-type N-glycan with no terminal galactose residue
M6	Oligomannose N-glycan containing 6 mannosyl residues
M5	Oligomannose N-glycan containing 5 mannosyl residues

run	L234F	H268Q	K274Q	Y296F	A327G	A330S	P331S
1	IgG1	IgG1	IgG1	IgG1	IgG1	IgG1	IgG1
2	IgG4	IgG1	IgG4	IgG4	IgG1	IgG1	IgG1
3	IgG1	IgG1	IgG4	IgG4	IgG4	IgG1	IgG1
4	IgG1	IgG1	IgG1	IgG4	IgG4	IgG1	IgG4
5	IgG4	IgG4	IgG1	IgG1	IgG1	IgG4	IgG4
6	IgG1	IgG4	IgG1	IgG1	IgG4	IgG4	IgG1
7	IgG4	IgG1	IgG1	IgG1	IgG4	IgG1	IgG4
8	IgG4	IgG1	IgG4	IgG1	IgG1	IgG4	IgG1
9	IgG4	IgG4	IgG4	IgG4	IgG4	IgG4	IgG4
10	IgG1	IgG4	IgG4	IgG1	IgG1	IgG1	IgG4
11	IgG4	IgG4	IgG4	IgG1	IgG1	IgG1	IgG1
12	IgG1	IgG1	IgG1	IgG4	IgG1	IgG4	IgG4
13	IgG1	IgG4	IgG4	IgG4	IgG1	IgG4	IgG1
14	IgG1	IgG1	IgG4	IgG1	IgG4	IgG4	IgG4
15	IgG4	IgG1	IgG1	IgG4	IgG4	IgG4	IgG1
16	IgG4	IgG4	IgG1	IgG4	IgG1	IgG1	IgG1

Supplementary Table 2. Design of a panel of 16 Design of Experiments combination mutant Fc hexamers. Each run represents an individual Fc hexamer construct with a combination of mutations. Each of the seven divergent CH2 residues in each mutant were allocated either the IgG1 amino acid (i.e. residue number prefix) or the IgG4 amino acid (i.e. residue number suffix) as indicated. Allocations per run are indicated in red for IgG1 or blue for IgG4.

Isotype	Mutation	Forward oligo	Reverse oligo
IgG1	**P331S**	CAAAGCCCTCCCAGCCAGCATCGAGAAAACCATC	GATGGTTTTCTCGATGCTGGCTGGGAGGGCTTTG
	**A330S**	TCTCCAACAAAGCCCTCCCAAGCCCCATCGAGA	TCTCGATGGGGCTTGGGAGGGCTTTGTTGGAGA
	**A327G**	GCGGGAGGAGCAGTTCAACAGCACGTACC	GGTACGTGCTGTTGAACTGCTCCTCCCGC
	**Y296F**	GCGGGAGGAGCAGTTCAACAGCACGTACC	GGTACGTGCTGTTGAACTGCTCCTCCCGC
	**K274Q**	CGAAGACCCTGAGGTCCAGTTCAACTGGTACGT	ACGTACCAGTTGAACTGGACCTCAGGGTCTTCG
	**H268Q**	TGGACGTGAGCCAGGAAGACCCTGAGG	CCTCAGGGTCTTCCTGGCTCACGTCCA
	**L234F**	GTGCCCAGCACCTGAATTCCTGGGGG	CCCCCAGGAATTCAGGTGCTGGGCAC
	**A330S P331S**	CTCCAACAAAGCCCTCCCAAGCAGCATCGAGAAAACCATCTCC	GGAGATGGTTTTCTCGATGCTGCTTGGGAGGGCTTTGTTGGAG
	**A327G P331S**	GTCTCCAACAAAGGCCTCCCAGCCAGCATCGAGAAAACC	GGTTTTCTCGATGCTGGCTGGGAGGCCTTTGTTGGAGAC
	**A327G A330S**	AAGGTCTCCAACAAAGGCCTCCCAAGCCCCATCGAGAAAAC	GTTTTCTCGATGGGGCTTGGGAGGCCTTTGTTGGAGACCTT
	**A327G A330S P331S**	GCAAGGTCTCCAACAAAGGCCTCCCAAGCAGCATCGAGAAAACCATCTC	GAGATGGTTTTCTCGATGCTGCTTGGGAGGCCTTTGTTGGAGACCTTGC
IgG4	**S331P**	AGGGACTGCCAAGCCCCATCGAGAAGACC	GGTCTTCTCGATGGGGCTTGGCAGTCCCT
	**S330A**	CAACAAGGGACTGCCAGCCTCCATCGAGAAGACC	GGTCTTCTCGATGGAGGCTGGCAGTCCCTTGTTG
	**G327A**	GGTCAGCAACAAGGCACTGCCAAGCTCCA	TGGAGCTTGGCAGTGCCTTGTTGCTGACC
	**F296Y**	GCCACGAGAGGAGCAGTATAACTCAACCTACCGTGT	ACACGGTAGGTTGAGTTATACTGCTCCTCTCGTGGC
	**Q274K**	AGGAAGACCCTGAGGTCAAGTTCAACTGGTATGTG	CACATACCAGTTGAACTTGACCTCAGGGTCTTCCT
	**Q268H**	TAGTGGATGTTAGCCATGAAGACCCTGAGGTCC	GGACCTCAGGGTCTTCATGGCTAACATCCACTA
	**F234L**	CCAGCTCCTGAGTTACTTGGCGGTCCTTC	GAAGGACCGCCAAGTAACTCAGGAGCTGG
	**S330A S331P**	TGGTCTTCTCGATGGGGGCTGGCAGTCCCTTGTTGCTG	CAGCAACAAGGGACTGCCAGCCCCCATCGAGAAGACCA
	**G327A S331P**	GTCAGCAACAAGGCACTGCCAAGCCCCATCGAGAAGAC	GTCTTCTCGATGGGGCTTGGCAGTGCCTTGTTGCTGAC
	**G327A S330A**	AGGTCAGCAACAAGGCACTGCCAGCCTCCATCGAGAAGAC	GTCTTCTCGATGGAGGCTGGCAGTGCCTTGTTGCTGACCT
	**G327A S330A S331P**	CAAGGTCAGCAACAAGGCACTGCCAGCCCCCATCGAGAAGACCATT	AATGGTCTTCTCGATGGGGGCTGGCAGTGCCTTGTTGCTGACCTTG

Supplementary Table 3. Oligonucleotide primers used for Design of Experiments panel and single site mutagenesis.

Supplementary Methods

Isolated neutrophil cytokine release assays

Blood from healthy human volunteers was collected into lithium heparin vacutainers (BD). Neutrophils were isolated by density gradient centrifugation with Ficoll Paque Plus (Amersham Biosciences). Briefly, 10 mL of blood was diluted 1:1 with RPMI 1640 (Gibco) and carefully layered onto 20 mL Ficoll Paque Plus. Cells were centrifuged for 30 min at 470 G. Neutrophils were collected and the contaminating erythrocytes were lysed in erythrocyte lysis buffer. Neutrophils were resuspended in MEM supplemented with 10 % FCS and plated at 5 x 10^5 / well in a 96-well round bottom tissue culture plate (Costar). Neutrophils were incubated with Fc hexamers at 10 μg/mL for 0, 6 or 24 h at 37 ^oC, 5 % CO_2, 100% humidity and supernatants collected for cytokine analysis by MSD multiplex.

Glycan analysis

Purified samples of Fc hexamer (100 μg) were denatured with 8 M urea / 55 mM Tris-HCl pH 8.0 and free thiols were capped with 22 mM iodoacetamide (IAM) for 60 min at 37 ^oC. Buffer was exchanged for 6 M urea / 50 mM Tris-HCl pH 8.0 before digesting with LysC/trypsin mix (Promega) for 3 h at 37 ^oC. The urea concentration was further reduced 5-fold by dilution and the digestion was continued overnight. Peptides were desalted with Waters Oasis HLB cartridges, dried using a centrifugal evaporator then reconstituted in 0.2 % formic acid (solvent A). Samples (7.5 μL, ~7 μg) were loaded at 150μL/min onto a 2.1 x 150mm C18 column (Waters 1.7u C18 PST 300A) equilibrated with 0.2 % formic acid at 40 ^oC. Peptides were eluted by a 60 min gradient to 50 % solvent B (4:4:1 acetonitrile: 1-propanol: water / 0.2 % formic acid) into a Waters Xevo mass spectrometer operated in MSE +ve-ion mode. MSE data, which consists of alternating scans of low and high collision energy, was collected over the range 100 - 1900 m/z. Digests were reduced by adding 10 mM Tris(hydroxypropyl)phosphine (THP) solution and incubating for >1 hat room temperature. Reduced samples were then re-analyzed. Waters BiopharmaLynx™ (BPL) was used for sequence analyses. Glycan profiles were determined from the peak areas of the various glycopeptide isoforms detected in the digests.
